# Risk-Sensitive Machine Learning for Financial Decision Modeling Under Imbalanced Data: Evidence from Bank Telemarketing

**DOI:** 10.3390/e28030354

**Published:** 2026-03-21

**Authors:** Bowen Dong, Xinyu Zhang, Yang Liu, Tianhui Zhang, Xianchen Liu, Lingmin Hou, Lingyi Meng, Zhen Guo, Aliya Mulati

**Affiliations:** 1 School of Electrical Automation and Information Engineering, Tianjin University, Tianjin 300072, China; 2 Department of Computer Science, Rochester Institute of Technology, Rochester, NY 14586, USA; 3 Department of Computer Science, University of Miami, Coral Gables, FL 33146, USA; 4 Department of Electrical and Computer Engineering, Northeastern University, Boston, MA 02115, USA; 5 Department of Electrical and Computer Engineering, Florida International University, Miami, FL 33174, USA; 6 School of Computing and Information, University of Pittsburgh, Pittsburgh, PA 15260, USA; 7 Department of Mechanical Engineering, Colorado State University, Fort Collins, CO 80523, USA

**Keywords:** machine learning, financial decision modeling, difficulty in minority-class identification under imbalance, imbalance modeling, class imbalance, risk-sensitive learning, interpretability

## Abstract

Bank telemarketing campaigns often experience low subscription rates due to customer heterogeneity and severe class imbalance, which pose challenges for reliable predictive modeling. This study investigates a data-driven approach that integrates synthetic minority oversampling and cost-sensitive learning to improve the prediction of telemarketing outcomes. Experiments are conducted using the Portuguese Bank Marketing dataset, comprising 41,188 instances with a positive response rate of 11.3%. Eight machine learning models are evaluated under a unified preprocessing pipeline and five-fold stratified cross-validation, including Logistic Regression, Decision Tree, Random Forest, and Ensemble methods. The results show that Ensemble models, particularly CatBoost, XGBoost, and LightGBM, achieve improved performance compared with traditional baselines, with notable gains in minority-class recall and overall discrimination ability. The best-performing model attains an F1-score of 0.540, a recall of 0.812 for the positive class, and a ROC–AUC of 0.908. To enhance interpretability, SHAP-based analysis is applied to quantify feature contributions, identifying campaign duration, previous contact outcomes, and selected macroeconomic indicators as key predictors. These findings indicate that combining resampling strategies with cost-sensitive optimization provides a robust and transparent approach for learning from imbalanced telemarketing data, thereby supporting reproducible and data-driven financial decision-making by explicitly addressing difficulty in minority-class identification under imbalance and class imbalance under cross-entropy training in imbalanced banking data.

## 1. Introduction

### 1.1. Research Background

In modern banking operations, telephone communication is an important channel for handling business [1]. Marketing activities that adopt telephone communication often have a success rate of less than 15%, which leads to an imbalance in resource allocation and an increase in operating costs [2]. Accurately predicting whether a client will subscribe to a term deposit has thus become an essential task for improving marketing effectiveness and optimizing decision support in financial institutions [3].

In recent years, the growing amount of structured customer data has made it possible to use data-driven predictive analytics to improve telemarketing results [4]. With demographic, behavioral, and macroeconomic variables, machine learning models can find hidden links between customer traits and subscription behavior [5]. But the predictive ability of these models is still limited by several long-standing problems [6].

First, class imbalance is a major issue in telemarketing data, because positive cases (successful subscriptions) make up only a small part of all contacts [7]. Standard classifiers often lean toward the majority class. They show high overall accuracy but low sensitivity to the minority class, which is the group most important for marketing decisions [8]. Second, customer differences create nonlinear and high-dimensional patterns that simple models cannot capture well [9]. Third, even though Ensemble and deep learning models have strong predictive power, their low interpretability makes it hard to ensure transparency and business use in regulated financial settings [10].

In this paper, we use the term class imbalance under cross-entropy training in an operational sense: it refers to cross-entropy (negative log-likelihood) asymmetry across classes under a skewed empirical prior, which manifests as disproportionate gradient mass being allocated to the majority class during training. This is distinct from maximizing Shannon entropy of the class distribution; our focus is the loss-level entropy (cross-entropy) and its class-conditional contributions.

### 1.2. Related Work

In the context of data-driven banking analytics, understanding past research is important to place the current study within the existing literature. This section reviews earlier work in three related areas: (1) predictive analytics for bank telemarketing, (2) learning from imbalanced marketing data, and (3) interpretability and explainable AI in financial decision-making. By combining findings from these areas, this section shows how telemarketing prediction has developed and points out the main gaps that lead to the proposed framework.

Telemarketing prediction has long been a common use case for data mining and machine learning in the banking industry [11]. Early studies mainly used statistical models, such as Logistic Regression, discriminant analysis, and probit models, to measure how socio-demographic and behavioral variables affect customer responses [12]. These methods gave clear interpretability, but they could not model nonlinear patterns or high-dimensional feature interactions that appear in real banking data [13,14,15].

However, even with these improvements, many studies judged models mainly by overall accuracy [16,17,18,19,20,21,22,23,24,25,26]. This ignores the class imbalance that affects performance and the interpretability needed for real banking use.

One major challenge in telemarketing analytics is the strong imbalance between successful and unsuccessful campaign outcomes. Only about 10–15% of contacted customers accept a term deposit offer, while most decline [27]. This imbalance causes conventional ML algorithms to minimize total error by predicting the majority class, thus achieving deceptively high accuracy but poor recall for the minority class [28]. As a result, models become practically ineffective for targeting the very customers that drive marketing profit.

To address this issue, research on imbalanced learning has progressed along two complementary paths:

(a) Data-level approaches. These methods modify the class distribution prior to model training. Over-sampling techniques such as the Synthetic Minority Over-sampling Technique (SMOTE) generate artificial minority samples by interpolating between neighbors, enriching the minority region [29]. Conversely, under-sampling techniques remove selected majority instances to reduce imbalance, though at the risk of losing information. Hybrid variants, such as SMOTE + Tomek Links or SMOTEENN, attempt to combine both strengths by simultaneously enriching minority data and cleaning noisy examples [30].
(b) Algorithm-level approaches. Rather than altering data, these methods adapt the learning process itself. Cost-sensitive learning assigns higher misclassification penalties to the minority class, influencing the optimization objective [31]. Ensemble-based techniques, including Balanced Random Forest and Easy Ensemble, construct multiple balanced subsets and aggregate weak learners to improve minority recall and reduce variance [32]. More recently, boosting algorithms such as XGBoost and LightGBM have incorporated built-in imbalance parameters, providing a flexible and scalable means of bias control [33].

Although such methods have been widely applied in credit scoring, fraud detection, and customer churn analysis, their systematic evaluation in telemarketing contexts remains scarce. In particular, few studies have compared both data-level and algorithm-level strategies within a unified pipeline that considers not only predictive performance but also business interpretability [34,35,36].

To address this issue, researchers have proposed model-agnostic interpretability methods such as LIME and SHAP [37]. SHAP is based on cooperative game theory and calculates feature contributions through Shapley values. It ensures both local accuracy and global consistency [38]. These methods have been applied to credit risk, customer retention, and fraud detection scenarios, providing auditable explanations. Although increasingly attracting attention, the application of explainability in telemarketing prediction is still rather limited. Many existing studies treat interpretability as a post hoc attachment instead of embedding it within the model design itself [39].

Recent studies have emphasized the integration of predictive modeling and interpretability in financial systems, arguing that model transparency is not merely a post hoc requirement but a structural component of responsible financial decision-making. For instance, recent work in data science and financial engineering has demonstrated how explainable machine learning frameworks can bridge predictive performance and regulatory compliance in financial forecasting and risk analysis contexts [40,41]. These studies highlight the growing need for interpretable architectures that embed explanation mechanisms within model evaluation rather than treating them as auxiliary analytical tools.

To make the positioning explicit, Table 1 qualitatively compares representative studies on the Portuguese Bank Marketing dataset along imbalance handling, evaluation protocol, and decision-oriented interpretability.

As shown in Table 1, prior work typically studies imbalance handling or explainability in isolation, whereas our framework explicitly unifies cost-aware cross-entropy, strict like-for-like ablations, and decision-oriented SHAP analysis.

They rarely discuss how model explanations translate into concrete marketing actions such as resource allocation or customer-group differentiation [42].

In summary, prior research in bank telemarketing has moved from simple statistical methods to more advanced ML models, but problems with class imbalance and interpretability still make real use difficult [43]. Both imbalance learning and explainable AI have been studied on their own, but using them together in one practical framework is still not well developed [44]. The next section therefore introduces the dataset, preprocessing procedures, and methodological design adopted in this study, detailing how imbalance correction and interpretability are embedded throughout the analytical pipeline. Existing macro-financial literature has long documented counter-cyclical savings behavior under labor market imbalance, reinforcing the need to incorporate macroeconomic indicators into predictive financial modeling [45,46].

We acknowledge that oversampling, class weighting, and Ensemble learners have been extensively studied. Our contribution is therefore not a new imbalance algorithm, but a controlled and decision-aligned framework that makes three aspects explicit: (i) an operational information-theoretic weighting strategy (cost-aware cross-entropy) that formalizes “entropy-aware risk regulation”; (ii) a strict like-for-like evaluation with ablations that isolates the marginal utility of each imbalance-handling component under identical preprocessing and validation; and (iii) an interpretability-to-decision bridge, where SHAP-based explanations are structured to support actionable campaign design rather than used as isolated plots.

### 1.3. Article Contributions

We operationalize the proposed ‘entropy-aware’ risk regulation by minimizing a class-weighted cross-entropy and present an information-theoretic interpretation, where class-dependent costs reshape the effective training distribution and gradient allocation. The model complexity term is treated as a standard regularizer and is conceptually separated from the cross-entropy component.

From an information-theoretic perspective, this study reframes class imbalance in bank telemarketing as a manifestation of difficulty in minority-class identification under imbalance and class imbalance under cross-entropy training, where rare subscription events represent low-frequency but high-information outcomes. By integrating resampling and cost-sensitive learning, the proposed framework can be interpreted as an entropy-aware risk regulation mechanism that redistributes the influence of imbalanced decision outcomes during model training. This perspective complements conventional performance-oriented analyses by explicitly linking predictive modeling with class imbalance under cross-entropy training management in financial decision-making.

This study does not claim novelty in individual algorithmic components (e.g., SMOTE, class weighting, or standard Ensemble learners), which are well established. Instead, the novelty lies in a rigorously operational and decision-oriented integration of these components, supported by an explicit information-theoretic formulation and a strict evaluation protocol. Specifically, we contribute:

(1) We provide an information-theoretic interpretation of class-weighted cross-entropy and clarify its relationship to cost-sensitive learning under class imbalance.

(2) Strict fairness protocol and ablation: under the same preprocessing and validation pipeline, we conduct controlled comparisons between data-level strategies (SMOTE), algorithm-level strategies (cost-sensitive weighting), and their combinations, including “no-imbalance-handling” baselines to isolate the incremental value of each component.

(3) Decision-oriented model selection and interpretability: beyond performance reporting, we link recall-priority and risk-sensitive decision strategies to model selection and provide structured SHAP explanations at global/interaction/local levels to translate model behavior into actionable campaign insights.

(4) Empirical performance improvement: It shows clear gains over classical models. The Ensemble methods raise minority recall by more than 25% and reach ROC–AUC values up to 0.91, showing the strength of the hybrid strategy.

(5) Practical decision implications: The framework provides useful insights for customer targeting and campaign planning, supporting transparent and data-driven decisions in the financial sector.

(6) We formalize a cost-weighted cross-entropy by deriving a cost-aware cross-entropy from an explicit business cost matrix ($C_{\mathrm{FN}},C_{\mathrm{FP}}$), provide its information-theoretic interpretation, and explain the mechanism via gradient reweighting for rare positives.

The remainder of this paper is organized as follows:

Section 2 reviews related studies on predictive modeling, imbalance learning, and explainable AI in marketing analytics.

Section 3 describes the dataset, preprocessing steps, feature engineering, and imbalance-handling methods.

Section 4 introduces the modeling approaches, including baseline classifiers, Ensemble learners, and validation strategies.

Section 5 presents the experimental results, interpretability analysis, and behavioral insights.

## 2. Data Preprocessing and Feature Engineering

**Figure 1:**
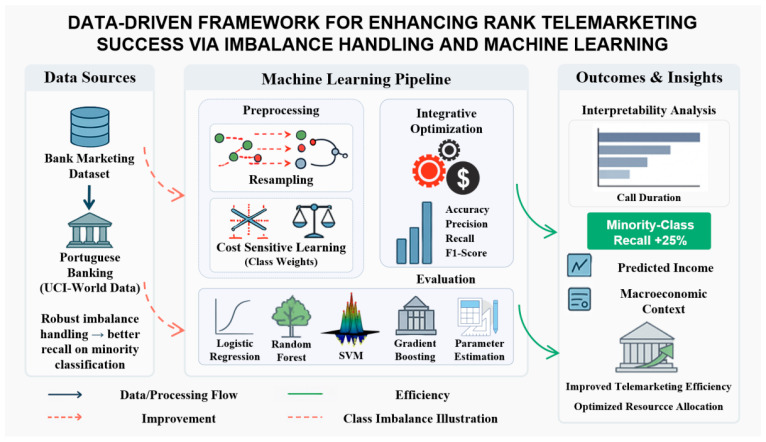
Overall framework of the data-driven telemarketing prediction system.

This section describes the approach for the framework that uses data to provide predictions in activities involving contact with individuals by telephone for marketing purposes. The process that combines all steps from obtaining initial information to developing features and addressing problems relating to differences in class frequencies appears in Figure 1. The process starts with gathering information from the Portuguese Bank Marketing Dataset and follows with preparing the data using steps that include removing errors, changing categories to numbers, and adjusting values to similar ranges.

To address the substantial difference between classes, methods that change the sample and approaches that make the learning process focus more on the class with less representation are both applied before developing the models. Following this, multiple approaches using methods that allow systems to learn from data are adjusted in specific ways and examined using measures that include precision, the measure of finding all relevant cases, the measure combining precision with finding cases, and the measure of area under the curve showing performance. The part on the right of the framework focuses on making the model clear to understand, examining how it can be interpreted, and considering the larger context relating to business and economics, showing how outputs from the model can support decisions in actual business situations and improve how resources are used in operations.

### 2.1. Dataset Description

The empirical analysis in this study uses the Bank Marketing Dataset, collected from a Portuguese financial institution and released publicly by Moro et al. (2014) [2]. The dataset contains outcomes of direct telemarketing campaigns promoting term-deposit products from 2008 to 2010. Each record corresponds to one client contact, and the dataset includes 41,188 instances with 20 input variables and one binary target variable that indicates whether the client subscribed to a term deposit (“y = yes/no”). These variables provide description of the background of clients in relation to socio-demographic factors, the operational features of the campaign, and indicators of the macroeconomic context that show the financial conditions present during contact.

Temporal validity and scope. We acknowledge that the Portuguese Bank Marketing data were collected in an earlier period (2008–2010) and that banking environments and consumer behaviors may evolve substantially over time (e.g., post-COVID digital adoption and policy shifts). Accordingly, the goal of this study is not to claim that a model trained on this historical dataset will remain optimal in today’s operational setting. Rather, we use this widely adopted public benchmark to develop and validate a reproducible, decision-oriented imbalance-aware framework (resampling + cost-aware learning + thresholding/calibration + SHAP-based decision support). The framework is intended to be retrained and recalibrated when deployed in a new time period or institution.

As summarized in Table 2, this group of variables includes socio-demographic factors (such as age, job, marital status, education), financial indicators (housing loan, personal loan, default history), and behavioral attributes derived from telemarketing interactions (contact duration, day of week, and month of contact). These variables jointly capture both who the client is and how they respond within the campaign process.

**Figure 2:**
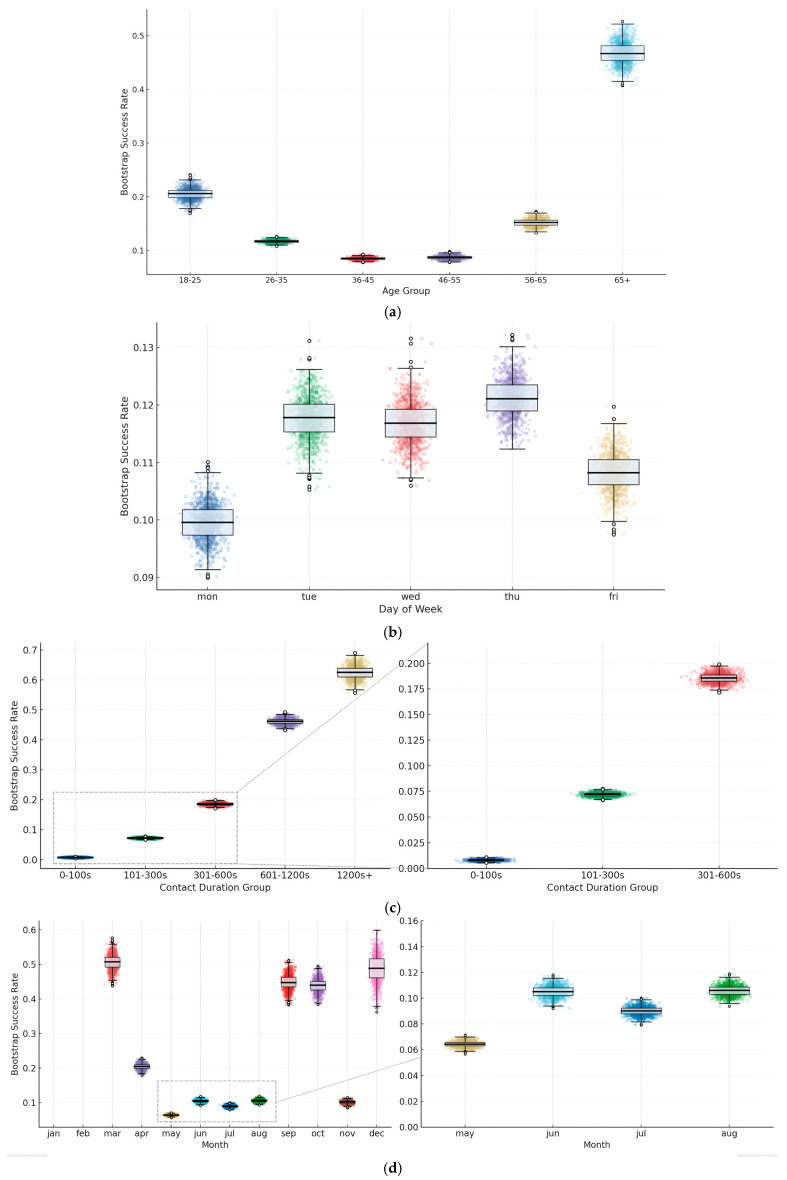
Exploratory analysis of client-level and behavioral attributes affecting subscription. (**a**) Bootstrap success rate distribution by age group. (**b**) Bootstrap success rate distribution by day of week. (**c**) Bootstrap success rate distribution by contact duration. (**d**) Bootstrap success rate distribution by month.

Figure 2 gives the overall picture of how telemarketing outcomes vary by some key client and campaign attributes. Cross-sectional differences in the weekdays exhibit that calls on Thursday and Friday are slightly more successful than those on the other days of the week, showcasing some effect of timing. Call duration is strongly positively related to success, with very short interactions seldom converting and long conversations much more likely to subscribe. Monthly seasonality displays peaks in March, September, and December and weaker months in the middle of the year. These patterns taken together give an indication that demographic factors, timing, and seasonal dynamics influence the effectiveness of campaigns and offer important cues for modeling and strategy design.

Campaign-related features describe how the telemarketing campaign is carried out, including time, frequency, and context of client contact. Together with demographic variables, these features are used to explain channels of communication, interaction history, and operational decisions that define campaign outcomes. Table 3 summarizes main variables related to the campaign execution using categorical and numerical indicators.

**Figure 3:**
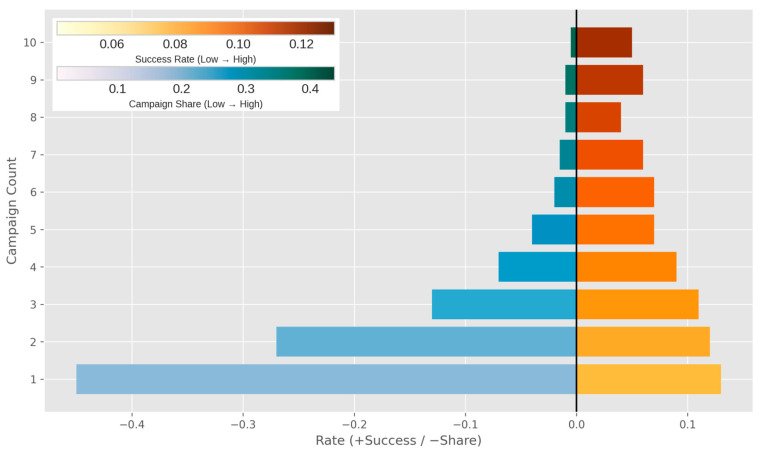
Campaign contact frequency and success rate distribution.

The data in Figure 3 show that the probability of positive outcomes reaches the main level during the initial contacts that occur, with a rate of approximately thirteen percent. This probability then shows a pattern of decrease with each contact that follows. The rate falls to a level below six percent when the total number of calls exceeds seven. The distribution that Panel b presents shows a pattern that differs substantially from an even distribution. This distribution indicates that most individuals in the study receive contact fewer than three times.

These measures include the rate of change in employment, the measure of confidence by individuals purchasing products, and the rate for loans at three months in the Euribor system, which together provide indication of the financial conditions in Portugal during the period examined. The variable y shows if an individual obtained a deposit product with a specific time period, and this forms a classification task with two categories that shows substantial differences between categories, with only approximately eleven percent of cases in the positive category. Table 4 presents the economic measures and the outcome variable that the study examines.

**Figure 4:**
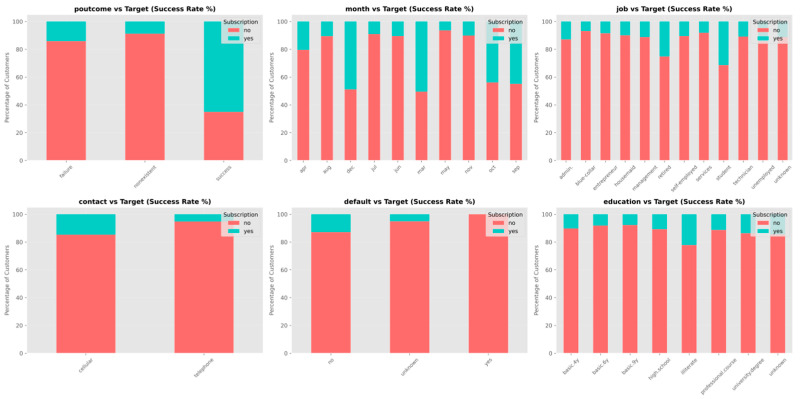
Relationship between categorical attributes and subscription outcome.

The analysis examining different categories reveals that features relating to previous behavior and features relating to demographic characteristics both affect outcomes for obtaining the product in Figure 4. These findings support the use of variables relating to individual characteristics and variables relating to the approach used in the process of developing the model that provides predictions, and this provides a foundation examining features for the following stages that involve representing features in forms that the model uses and developing the model using the data.

### 2.2. Data Preprocessing Procedures

The process of preparing the data provides necessary steps for creating standard forms for variables, representing these variables in forms that the model uses, and preventing the problem of using information that appears in the outcome during the process of developing the model, and these steps are necessary because the data include a large number of dimensions, substantial variation in the types of variables, and substantial differences in the frequency of the two categories, with eleven point three percent of cases in the positive category. The study applies all changes to the form of variables using only the portion of data used for developing the model and then applies these changes to the portion of data used for assessment, which provides the appropriate method for the study.

#### 2.2.1. Data Cleaning and Integrity Check

Before modeling, the data should be checked for validity and consistency. Preprocessing steps involve removing duplicate entries, dealing with missing values by imputing where necessary, checking data types are correct, and ensuring categorical variables such as occupation, education, and marital status use consistent labeling. Numerical variable outliers are not removed, except where obviously due to data-entry errors, in order not to affect real economic dispersion.

**Figure 5:**
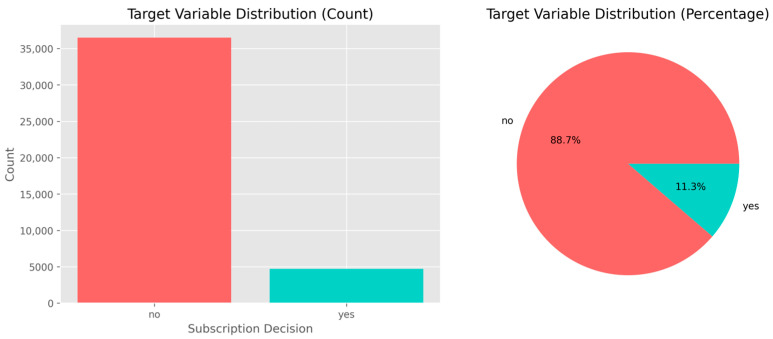
Class imbalance of target variable distribution.

After feature integrity was assured, the target variable, identifying whether the client subscribed to a term deposit, was examined to demonstrate an additional structural problem inherent in the dataset. As shown in Figure 5, the class distribution is highly imbalanced: 11.3 percent of the contacts resulted in a subscription (“Yes”), while 88.7 percent were non-subscriptions (“No”). This class imbalance means that if the data is used without proper modifications, typical machine learning models will tend to overpredict the majority class, yielding poor recall of the minority class.

Figure 5 depicts the marked imbalance between positive (“Yes”) and negative (“No”) outcomes. Given that the minority class encompasses only 11.3 percent of all records, modeling becomes necessary with a focus on imbalance. Verification of this distribution links the exploratory analysis with the preprocessing stage in confirming that the data are structurally sound while class imbalance is highlighted as the central challenge that motivates the use of resampling and feature-standardization procedures.

Missing Value Handling and Outlier Treatment. A systematic data-cleaning protocol was implemented to ensure reproducibility and prevent information leakage. The original dataset contains minimal missing values (<0.5% across all features). For categorical variables, missing entries were imputed using the mode computed within each training fold. For numerical variables, median imputation was applied to preserve robustness against skewed distributions.

All imputation statistics were calculated exclusively from the training data and subsequently applied to the validation and test folds, thereby avoiding data leakage.

Duplicate records were removed when exact feature matches occurred. Regarding outlier treatment, no winsorization or trimming was applied to numerical variables, since extreme values (e.g., long call durations or macroeconomic fluctuations) reflect genuine economic dispersion inherent in telemarketing operations.

Only values identified as clear data-entry errors were excluded. This standardized cleaning procedure ensures transparency and reproducibility for subsequent modeling stages.

After removing zero exact duplicate records, the final dataset retained 41,188 instances. No rows were discarded due to missing values.

The class distribution after cleaning remained 36,548 negative samples (88.73%) and 4640 positive samples (11.27%).

#### 2.2.2. Variable Encoding and Normalization

The dataset has 11 categorical variables of the following nature: job type, marital status, education level, contact channel, month, and prior campaign outcomes. Encoding for these needs different approaches.

Low-cardinality features have five or fewer categories: marital status, housing loan status, personal loan status, and contact type. They are one-hot encoded to retain interpretability and avoid imposing artificial ordinal structure.

Target encoding is applied for high-cardinality features, those with six or more categories: job, education. Every category is replaced by its smoothed estimated success probability:

(1) $$\mathrm{Enc}(c)\;=\;\frac{n_{c}\cdot p_{c}+\alpha \cdot p_{\mathrm{global}\;}}{n_{c}+\alpha }$$

where $n_{c}$ is the category frequency, $p_{c}$ is local success rate, and $p_{\mathrm{global}}$ is the overall campaign success rate.

Cyclical encoding for time-related categories. The variables month and day of week are encoded as cyclical features to preserve periodicity. For a time index t with period T, we use

(2) $$x_{\sin}=\sin(2\pi t/T)$$

(3) $$x_{\cos}=\cos(2\pi t/T)$$

Specifically, T = 12 for month (mapped to 1–12) and T = 5 for day of week (mapped to 1–5). If normalization is applied, the resulting numerical features are standardized after encoding using mean and standard deviation computed within each training fold, and then applied to validation/test folds to avoid leakage.

#### 2.2.3. Feature Engineering and Data Partitioning

To increase the strength of the data and examine patterns that relate to behavior and context, multiple features using domain logic were developed. These features follow from principles in telemarketing and financial work. The main focus was to provide indicators that show performance and allow interpretation. These indicators reflect how efficiently calls occur, how recent interactions are, and what trends appear in macroeconomic conditions. The approach bridges variables in raw form with meaning that relates to campaign management at a larger level.

First, a measure of contact efficiency was designed to show the average persuasive effect of each marketing interaction. It was defined as the ratio between the duration of the last successful call and the total number of contact attempts in the current campaign.

(4) $$R_{\mathrm{eff}\;}=\frac{t_{\mathrm{duration}}\;}{\;n_{\mathrm{campaign}}\;}$$

where $R_{\mathrm{eff}\;}$ denotes the contact efficiency ratio, duration represents the duration of the most recent call (in seconds), and campaign indicates the total number of calls made to a client within the ongoing campaign. A higher $R_{\mathrm{eff}\;}$ reflects greater persuasive yield per contact attempt and is empirically associated with a higher probability of customer conversion.

Recency variable handling. We do not introduce an additional engineered recency feature (e.g., an inverse transform of pdays), because such transformations are monotonic mappings of pdays and do not add new information beyond the original variable. Therefore, customer recency is represented directly by pdays (with 999 indicating no prior contact).

This indicator shows how changes in financial markets affect clients’ investment decisions. The indicator is defined as follows:

(5) $$M_{\mathrm{trend}\;}=\frac{{\;\mathrm{Euribor}}_{t}\;-{\;\mathrm{Euribor}}_{t-1}}{{\;\mathrm{Euribor}}_{t-1}}$$

where $M_{\mathrm{trend}}$ is the indicator of macroeconomic momentum. ${\;\mathrm{Euribor}}_{t}$ does represent the 3 month Euribor rate over the current campaign period with ${\;\mathrm{Euribor}}_{t-1}$ the same rate over the previous campaign.

**Figure 6:**
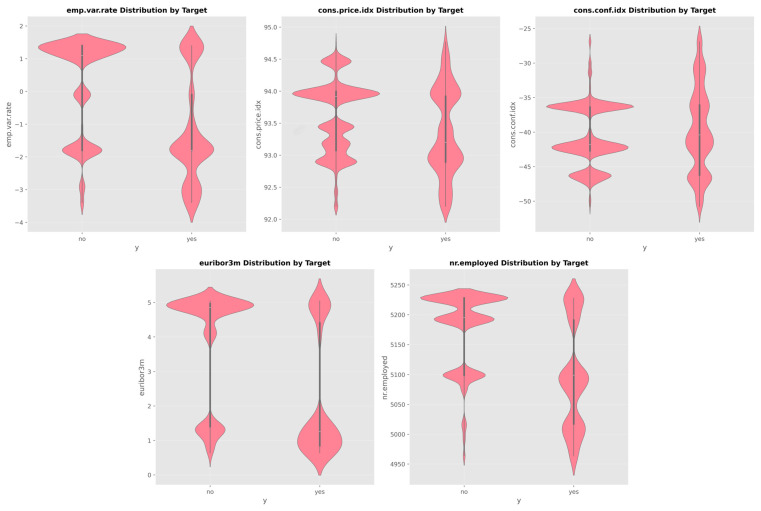
Distribution of macroeconomic indicators by target variable.

Figure 6 shows the analysis of five important macroeconomic indicators:

- Employment variation rate (emp.var.rate).
- Consumer price index (cons.price.idx).
- Consumer confidence index (cons.conf.idx).
- 3-month Euribor rate (euribor3m).
- Number of employees (nr.employed).

These indicators display clear distributional differences between subscription outcomes.

To check the ability of the model to generalize, we stratified random sample split the dataset into:

- Training set: 80% (32,950 samples).
- Testing set: 20% (8238 samples).

Following preprocessing, the dataset was converted into numbers and normalized, while the categorical variables were transformed as per the encoding methods. Correlation analysis showed that campaign-related attributes such as duration and previous outcomes have the strongest positive association with the target variable. Some macroeconomic variables, such as emp.var.rate and euribor3m, show negative correlation, which matches the idea that difficult economic conditions reduce interest in term deposits.

The fully preprocessed dataset, stored as an HDF5 file for model training (see Appendix A), provides a structured and standardized base for the next analysis steps.

### 2.3. Imbalance-Handling Techniques

This dataset exhibits significant class imbalance, which from an information-theoretic perspective corresponds to a highly skewed information distribution. In such settings, minority-class instances represent low-frequency but high-information events, while majority-class samples largely contribute redundant information, increasing imbalance in data-driven learning. To mitigate such data bias issues, this study employs a hybrid approach to handling imbalanced data. This combines resampling at the data level with cost adjustment at the algorithmic level. This dual strategy maintains model stability while enhancing sensitivity towards the minority cases.

#### 2.3.1. Data-Level Resampling

At the data level, synthetic oversampling methods were used to increase the size of the minority class through feature-space interpolation. This reduces imbalance before model training. Two common approaches—Synthetic Minority Oversampling Technique (SMOTE)—were applied to compare their effects. In SMOTE, each minority-class sample $x_{i}$ is paired with one of its k-nearest minority neighbor $x_{\mathrm{zi}}$, and a synthetic sample is generated between them:

(6) $$x_{\mathrm{new}}\;=\;x_{i}\;+\;\delta \times \left(x_{\mathrm{zi}}\;-\;x_{i}\right),\;\delta ∼U\left(0,1\right)$$

where $x_{\mathrm{new}}$ represents the generated synthetic instance, $x_{i}$ is an existing minority class sample, $x_{\mathrm{zi}}$ denotes one of its randomly selected minority neighbors, and δ is a random interpolation coefficient uniformly distributed in [0, 1]. This process effectively expands the decision region of the minority class in the feature space, alleviating overfitting caused by simple replication.

Building on this concept, it introduces adaptivity by generating more synthetic samples for minority points located in regions of higher learning difficulty. Let G denote the total number of synthetic examples to generate, and let $r_{i}$ represent the relative density of majority neighbors surrounding a minority instance $x_{i}$. Then, the number of synthetic samples assigned to $x_{i}$ is computed as

(7) $$G_{i}=\frac{r_{i}}{\sum_{j=1}^{N_{m}}\;r_{j}}\times G$$

where $G_{i}$ is the number of synthetic examples created for instance i, $N_{m}$ is the total number of minority samples, and $r_{i}$ quantifies the local imbalance ratio around $x_{i}$. In this formulation, instances with higher $r_{i}$, those surrounded by more majority samples-receive proportionally more synthetic reinforcement, directing model attention toward harder-to-learn regions of the feature space.

SMOTE was executed solely within the training folds of the cross-validation process to prevent data leakage. Their parameters, including the number of nearest neighbors k = 5 and the oversampling ratio (target minority proportion = 30%), were empirically optimized using validation F1-score as the selection criterion.

First, adversarial training may introduce instability, mode collapse, or distributional distortion, particularly in moderately sized datasets with mixed categorical–numerical features.

Second, GAN-generated samples lack the geometric interpretability inherent in interpolation-based methods such as SMOTE, which construct synthetic instances along observed minority-class manifolds. In regulated financial contexts, preserving traceability and distributional consistency is essential.

Third, incorporating GAN-based augmentation would introduce additional hyperparameter complexity, potentially confounding the comparative evaluation of downstream classifiers.

Future research may investigate adversarial data augmentation within entropy-regularized frameworks to further explore synthetic distribution learning in financial decision modeling.

In addition to the default configuration (k = 5, target minority proportion = 30%), we conducted a parameter sensitivity analysis by varying the SMOTE target ratio and the number of neighbors k within the cross-validation pipeline. Specifically, the target minority proportion was swept over {15%, 20%, 30%, 40%, 50%}, and k was evaluated over {3, 5, 7, 9, 11}. All resampling operations were performed exclusively on training folds to prevent information leakage.

Given the structured tabular nature of the dataset, which contains mixed categorical and numerical features, interpolation-based oversampling is particularly suitable.

The objective of this study is not to propose a new data augmentation algorithm, but to evaluate imbalance-handling strategies under a strictly controlled modeling pipeline.

SMOTE provides deterministic geometric interpolation within minority-class neighborhoods, allowing stable integration within cross-validation folds.

Therefore, SMOTE was selected as a transparent and controlled data-level strategy consistent with the study’s methodological focus.

Within each training fold, the minority class size increased from approximately 3712 to 9885 instances under the 30% target proportion setting (exact values vary slightly across folds due to stratification). To ensure that the choice of resampling strategy does not bias conclusions, we also report ablation results comparing resampling-only, cost-sensitive-only, and combined strategies.

#### 2.3.2. Algorithm-Level Cost-Sensitive Learning

While resampling methods rebalance data distributions, they may introduce noise or synthetic redundancy. To complement them, algorithm-level cost-sensitive learning modifies the optimization objective of classifiers to impose asymmetric penalties on misclassification errors. Let L(θ) denote the empirical loss function of a model parameterized by θ. In cost-sensitive learning, the loss is weighted by a class-dependent coefficient $w_{y_{i}}$ such that:

(8) $$L(\theta )=\Sigma_{n=1}^{N}w_{y_{i}}\;I(y_{i},\;{\hat{y}}_{i})$$

where $y_{i}$ is the true class label of sample i, ${\hat{y}}_{i}$ is its predicted probability, $I\left(y_{i},\;{\hat{y}}_{i}\right)$ denotes the base loss function (binary cross-entropy), and $w_{y_{i}}$ is the misclassification cost weight. Among them, the weight factor is defined as:

(9) $$w_{y_{i}}=\left\{\begin{array}{c} \;w_{p}=\frac{N_{\mathrm{neg}}}{N_{\mathrm{pos}}},\;if\;y_{i}=1\;(positive\;sample)\;\\ 1,\;if\;y_{i}=0\;(negative\;sample)\; \end{array}\right.$$

where $w_{p}$ can increase the penalty intensity for negative samples. $N_{\mathrm{neg}}$ and $N_{\mathrm{pos}}$ represent the number of negative and positive samples respectively. The effective contributions of these two types of samples to the gradient update will tend to be balanced.

To provide an information-theoretic motivation, suppose we wish each class to contribute equally in expectation to the optimization objective.

Let p(y) denote the empirical class prior. To equalize expected weighted contribution:

(10) $$E[w(Y)|(Y=y)]\propto 1\Rightarrow w(y)\propto \frac{1}{p(y)}$$

Since $p(y)\;\approx \;N_{y}/N$, this yields:

(11) $$w(y)\propto \frac{N}{N_{y}}$$

which corresponds to the adopted weighting scheme.

Moreover, the self-information (surprisal) of class y is:

(12) I(y) = −log p(y)

Both 1/p(y) and −log p(y) are monotonically increasing as p(y) decreases. Thus, inversefrequency weighting is consistent with allocating higher optimization emphasis to low-probability, high-information events.

In this formulation, misclassification costs can be interpreted as asymmetric information losses, and cost weighting serves to regulate the contribution of decision imbalance during model optimization.

#### 2.3.3. Explicit Cost Matrix Specification and Implementation

To eliminate heterogeneous tabular data regarding cost-sensitive learning, we explicitly specify the numerical form of the cost matrix and its implementation in all experiments.

Let $N_{\mathrm{pos}\;}$ and $N_{\mathrm{neg}\;}$ denote the numbers of positive (“yes”) and negative (“no”) samples in the training data, respectively. Based on the cleaned dataset, the class counts are: $N_{\mathrm{neg}\;}=36,548,\;N_{\mathrm{pos}\;}=4640$.

We adopt inverse-frequency class weighting. The positive-class weight is defined as

$$w_{pos}=\frac{N_{neg}}{N_{pos}}=\frac{36,548}{4640}\approx 7.88$$

and the negative-class weight is fixed to

$$w_{\mathrm{neg}\;}=1$$

This corresponds to the following cost matrix formulation:

$$C=\left(\begin{array}{cc} 0 & C_{FP}\\ C_{FN} & 0 \end{array}\right)=\left(\begin{array}{cc} 0 & 1\\ 7.88 & 0 \end{array}\right)$$

where $C_{FN}=7.88$ penalizes false negatives more heavily than false positives.

No additional business-driven or manually tuned cost matrix was introduced. The weight ratio was fixed to the inverse class frequency and kept constant across all experiments to avoid introducing additional degrees of freedom.

The cost-sensitive scheme was implemented consistently in all classifiers supporting class weighting:

- Logistic Regression and Linear SVM: implemented via the class_weight parameter using {pos: 7.88, neg: 1}.
- Random Forest: implemented using class_weight = ‘balanced’, which internally applies inversefrequency weighting equivalent to the above ratio.
- XGBoost: implemented via scale_pos_weight = 7.88.
- LightGBM: implemented via scale_pos_weight = 7.88.
- Transformer model: trained using weighted binary cross-entropy, where positive samples were multiplied by 7.88 and negative samples by 1 during loss computation.

Unless otherwise specified, all reported cost-sensitive results in Section 4 employ this inversefrequency weighting scheme. The class-weighted cross-entropy used in this study corresponds directly to the above class weights.

#### 2.3.4. Cost-Weighted Cross-Entropy and Information-Theoretic Interpretation

This objective is mathematically equivalent to standard class-weighted cross-entropy under weighted empirical risk minimization.

Our contribution is to provide an explicit information-theoretic interpretation and to evaluate this weighting scheme under a strictly controlled and reproducible experimental protocol, rather than proposing a new entropy formulation or optimization framework.

Let y∈{0,1} and $p_{\theta }(x)=P(y=1|x)$. Standard binary cross-entropy is defined as:

(13) $$L_{\mathrm{CE}}(\theta )\;=\;-E\left[y\cdot \log p_{\theta }(x)+(1-y)\log\left(1-p_{\theta }(x)\right)\right]$$

In telemarketing decision-making, false-negative errors incur higher opportunity cost than false positives. To explicitly encode asymmetric business risk, we define a cost-weighted cross-entropy:

(14) $$L_{\mathrm{RS}}(\theta )\;=\;-E\left[\alpha y\cdot \log p_{\theta }(x)+(1-\alpha )(1-y)\log\left(1-p_{\theta }(x)\right)\right]$$

where $\alpha \;=\frac{C_{\mathrm{FN}}}{C_{\mathrm{FN}}+C_{\mathrm{FP}}}$ is derived from inverse-frequency class weighting implemented in the experiments.

Let the weighted loss be defined as:

(15) $$L_{w}(\theta )\;={\;E}_{(x,y)∼p}\left[w(y)\left(-\log q_{\theta }(y\;|\;x)\right)\right]$$

Define a reweighted joint distribution:

(16) $$p_{w}(x,\;y)\;=\;\frac{w(y)\;p(x,\;y)}{Z},\;Z\;=\;E_{p}[w(Y)]$$

Then the loss becomes:

(17) $$L_{w}(\theta )\;=\;Z\cdot E_{(x,y)∼p_{w}}\left[-\log q_{\theta }(y\;|\;x)\right]$$

Using the cross-entropy decomposition:

(18) $$E_{p_{w}}\left[-\log q_{\theta }(Y|X)\right]\;=\;H_{p_{w}}(Y|X)+D_{\mathrm{KL}}\left(p_{w}(Y|X)\|q_{\theta }(Y|X)\right)$$

Thus, minimizing the weighted binary cross-entropy is equivalent to minimizing:

(19) $$D_{\mathrm{KL}}\left(p_{w}(Y|X)\|q_{\theta }(Y|X)\right)$$

under a reweighted data distribution $p_{w}$.

For positive instances (y=1), the gradient becomes:

(20) $$\frac{\partial L_{\mathrm{RS}}}{\partial p}\;=\;-\frac{\alpha }{p}$$

indicating that larger α amplifies the learning signal of rare positive samples. When $C_{\mathrm{FN}}=C_{\mathrm{FP}}$, the objective reduces to standard cross-entropy.

It is important to clarify that the loss re-interpretation does not attempt to maximize the Shannon entropy H(Y) nor enforce a uniform class distribution. Instead, class imbalance under cross-entropy training in this study refers to the uneven allocation of learning signal (risk mass) across classes under skewed empirical priors.

The optimization target is the conditional cross-entropy under the reweighted distribution $p_{w}$, which implicitly redistributes gradient contributions between classes rather than altering the intrinsic entropy of the label distribution.

For clarity, we do not claim a new entropy-based optimization framework; the loss used in our experiments is standard class-weighted cross-entropy, and the novelty is limited to interpretation and controlled empirical analysis.

#### 2.3.5. Integrated Imbalance-Handling

To provide consistent and scalable handling of class imbalance during the modeling process, an integrated imbalance-handling pipeline was built. The pipeline combines data-level resampling with algorithm-level cost-sensitive learning inside one cross-validation framework. This allows the models to use the extra diversity created by synthetic samples while still applying penalties for misclassified minority cases.

Let the training dataset after preprocessing be

(21) $$D={\left\{\left(x_{i},y_{i}\right)\right\}}_{i=1}^{N},y_{i}\in \{0,1\}$$

where $y_{i}=\;1$ denotes a successful subscription and $y_{i}=\;0$ otherwise.

The overall objective can therefore be formally expressed as the Entropy-Regularized Objective Function:

(22) $$\underset{\theta }{\min}\;L(\theta )\;=\;\frac{1}{N}\sum_{i=1}^{N}\;w_{y_{i}}\;I\left(f\;\left(x_{i}\;;\;\theta \right),{\;y}_{i}\right)\;+\;\alpha \Omega (\theta )$$

where $w_{y_{i}}$ is class weight, assigning higher penalty to minority-class errors; I(⋅) is loss function. For clarity, the entropy terminology in this study refers exclusively to cross-entropy and KL-divergence in the information-theoretic sense. The complexity control term αΩ(θ) is introduced solely to prevent overfitting and is conceptually independent from entropy. α controls Ω(θ) and is unrelated to γ/λ in XGBoost.

Therefore, entropy in this paper denotes predictive imbalance measured through cross-entropy, whereas regularization refers to model complexity control.

The class weight is defined as

(23) $$w_{y_{i}}=\left\{\begin{array}{c} \frac{N}{2N_{1}},y_{i}=1\\ \frac{N}{2N_{0}},y_{i}=0 \end{array}\right.$$

where $N_{1}$ and $N_{0}$ are the sample counts of the minority and majority classes, respectively. This weighting scheme balances gradient contributions across classes and is compatible with all tree-based Ensemble learners and linear models used in later sections.

The integrated imbalance-handling pipeline harmonizes synthetic resampling and cost-sensitive learning within a single, modular architecture. By maintaining this structure consistently across all experiments, the subsequent modeling and evaluation stages directly reflect algorithmic efficacy rather than artifacts of data imbalance or sampling variance while maintaining entropy-balanced gradient contributions across classes.

## 3. Modeling Approaches and Evaluation

This chapter outlines the general modeling framework used for predicting subscription outcomes in bank telemarketing campaigns. The models are built using the cleaned and rebalanced dataset described in Section 2.

### 3.1. Baseline Models

These models establish the performance reference for more complex Ensemble and neural architectures. Unless otherwise specified, the default setting uses SMOTE together with cost-sensitive weighting. To quantify the contribution of each component, we further conducted ablation experiments under three variants (resampling only, cost-sensitive only, and their combination).

In addition to conventional classifiers and gradient-boosting Ensembles, we further include high-quality baselines specifically designed for imbalanced learning: Balanced Random Forest (BRF), which balances bootstrap samples to reduce majority dominance, and EasyEnsemble (EE), which trains an Ensemble over multiple balanced subsets to improve minority recall while controlling variance. Moreover, we consider Focal Loss as an imbalance-aware class-weighted cross-entropy for neural models, which down-weights easy majority samples and emphasizes hard examples. All added baselines follow the same preprocessing and 5-fold stratified cross-validation protocol to ensure strict comparability.

Logistic Regression (LR). In the approach using assessment of likelihood for assignment to groups, the measure of likelihood that the individual provides a positive response, denoted as P with y equal to one given X, follows from a particular function:

(24) $$P(y=1|X)\;=\;\frac{1}{1+e^{-\left(\beta_{0}+\sum_{i=1}^{n}\;\beta_{i}x_{i}\right)}}$$

where $\beta_{i}$ indicates the measure relating to feature $x_{i}$. The process for finding parameter values involves reducing error in assignment while applying a method that limits parameter growth. This method provides stability in the process of finding values and prevents measures from showing excessive increase.

Decision Tree (DT). The Decision Tree uses a hierarchical approach to partition the feature space. At each internal node, the algorithm selects a feature $x_{j}$ and threshold t that maximizes the coverage:

(25) $$IG\left(x_{j},t\right)=H(D)-\sum_{k\in \{L,R\}}\;\frac{\left|D_{k}\right|}{\left|D\right|}H\left(D_{k}\right)$$

where $H\left(D_{k}\right)$ is the entropy of the observations directed to the left nd right segments, respectively. The aforementioned structure exhibits a lucid and intelligible path of decisions from the root to the corresponding leaf node. Hence, it is especially useful in interpreting analyses.

Random Forest (RF). A Random Forest builds an Ensemble of Decision Trees using bootstrapped samples and random feature subsets. The final prediction is given by:

(26) $$\hat{y}\;=\;\mathrm{mode}\left({\left\{h_{b}(X)\right\}}_{b=1}^{B}\right)$$

where B is the total number of trees and $h_{b}(X)$ represents the prediction of the $b^{\mathrm{th}}$ tree.

In this work, the Random Forest uses 200 estimators, a maximum depth of 12, minimum leaf size of 5, and bootstrap sampling. RF is resistant to noise and overfitting, but it may still be affected by class imbalance. Even so, it provides a strong baseline for comparison.

Support Vector Machine (SVM). The Support Vector Machine constructs an optimal separating hyperplane by maximizing the margin between positive and negative samples. Given training data ${\left\{\left(x_{i},y_{i}\right)\right\}}_{i=1}^{m}$, SVM solves the following optimization problem:

(27) $$\begin{array}{c} \underset{w,b,\xi_{i}}{\min}\;\frac{1}{2}{\left\|w\right\|}^{2}+C\sum_{i=1}^{m}\;\xi_{i}\\ \;s.t.\;y_{i}\left(w\cdot \phi \left(x_{i}\right)+b\right)\geq 1-\xi_{i},\;\xi_{i}\geq 0 \end{array}$$

where $\phi \left(x_{i}\right)$ is the feature mapping, C controls the penalty for misclassification, and $\xi_{i}$ are slack variables allowing soft margins. An RBF kernel is used to model nonlinear relationships. 

Regarding the above mentioned basic models, this article proposes an Imbalance-aware modeling pipeline, which is presented in Algorithm 1.
**Algorithm 1** Imbalance-aware modeling pipeline **Input:** $\mathrm{dataset}\;D\;=\;\{(x_{i},{\;y}_{i})\}$, model set M, number of folds K,  SMOTE parameters θ$,\;\mathrm{class}\;\mathrm{weights}\;(w_{+,}\;w_{-}$) **Output:** $\mathrm{selected}\;\mathrm{model}\;f_{*}$  **repeat**   **for** k = 1 to K **do**     Partition D$\;\mathrm{into}\;{D_{\mathrm{train}}}^{\left(k\right)}$$\;\mathrm{and}\;{D_{\mathrm{val}}}^{\left(k\right)}$     Preprocess both subsets $\;\;\;\;\mathrm{Apply}\;\mathrm{SMOTE}\;\mathrm{to}\;\mathrm{obtain}\;\left(X^{\left(k\right)},\;{\tilde{y}}^{\left(k\right)}\right)$     **for** each model m∈M do $\;\;\;\;\;\mathrm{Assign}\;\mathrm{class}\;\mathrm{weights}\;(w_{+},{\;w}_{-}$)      Train m$\;\mathrm{on}\;\left(X^{\left(k\right)},\;{\tilde{y}}^{\left(k\right)}\right)$$\;\mathrm{to}\;\mathrm{obtain}\;{f_{m}}^{\left(k\right)}$
$\;\;\;\;\;\mathrm{Compute}\;\mathrm{predictions}\;p\;=\;{f_{m}}^{\left(k\right)}({X_{\mathrm{val}}}^{\left(k\right)})$      **for** i = 1 to |p| **do**       **if** p[i] ≥ τ then $\;\;\;\;\;\;\;\;\;\overset{ˆ}{y}[i]\;=\;1$       **esle** $\;\;\;\;\;\;\;\;\overset{ˆ}{y}[i]\;=\;0$       **end if**        **end for**       Record the metric score for m    **end for**   **end for**  Compute AvgScore [m] for all m $\;\mathrm{Select}\;f_{*}\;=\;\mathrm{argmax}\{\mathrm{AvgScore}[m]\}$ **until** $f_{*}$ is stable

### 3.2. Advanced Ensemble Models

Baseline models provide interpretability and performance references, but their limited ability to capture complex nonlinear patterns and imbalance motivates the use of stronger Ensemble methods. Ensemble models combine multiple weak learners, usually Decision Trees, into a single predictive system that reduces bias and variance.

This section presents Gradient Boosting (GB), Extreme Gradient Boosting (XGBoost), Light Gradient Boosting Machine (LightGBM), and CatBoost as the main Ensemble algorithms used in the framework. A Transformer-based extension is also described briefly for completeness.

Gradient Boosting (GB). All boosting algorithms share the same conceptual foundation: an additive model that minimizes a differentiable loss function by sequentially fitting weak learners (typically shallow trees). The general form is given by

(28) $$F_{M}(x)=\sum_{m=1}^{M}\;\gamma_{m}h_{m}(x)$$

where $F_{M}(x)$ denotes the final Ensemble prediction, $h_{m}(x)$ is the m-th weak learner, and $\gamma_{m}$ is its learning weight determined via line search. At each iteration, a new learner is trained to approximate the negative gradient of the loss function with respect to current predictions:

(29) $$g_{i}^{\left(m\right)}\;=\;-\left[\frac{\partial I\left(y_{i},{\;F}_{m-1}\left(x_{i}\right)\right)}{\partial F_{m-1}\left(x_{i}\right)}\right]$$

where $g_{i}^{\left(m\right)}$ represents the pseudo-residual for sample i, I(⋅) is the differentiable loss (binary cross-entropy in this study), $y_{i}$ is the ground-truth label, and $F_{m-1}\left(x_{i}\right)$ is the prediction from the previous iteration. Each subsequent learner $h_{m}(x)$ minimizes the residual sum of squares:

(30) $$h_{m}(x)\;=\;\arg\;\underset{h}{\min}\;\sum_{i=1}^{n}\;{\left(g_{i}^{\left(m\right)}-h\left(x_{i}\right)\right)}^{2}$$

where followed by a global update of the Ensemble model.

Extreme Gradient Boosting (XGBoost). XGBoost enhances the basic gradient boosting framework through second-order Taylor expansion of the loss function, allowing the use of both first and second derivatives during optimization. The objective function is expressed as:

(31) $$L^{\left(t\right)}\;=\sum_{i=1}^{n}\;\left[g_{i}\;f_{t}\left(x_{i}\right)+\frac{1}{2}h_{i}{\;f}_{t}{\left(x_{i}\right)}^{2}\right]+\Omega \left(f_{t}\right)\;$$

where $g_{i}$ and $h_{i}$ denote the first and second derivatives of the loss with respect to the current prediction, and $\Omega \left(f_{t}\right)$ is the regularization term:

(32) $$\Omega \left(f_{t}\right)\;=\;\gamma T+\frac{1}{2}\lambda \|w{\|}^{2}$$

Here, T is the number of leaves in the tree, w the leaf weights, γ the leaf penalty term, and λ the L2 regularization coefficient. By explicitly penalizing model complexity, XGBoost prevents overfitting while improving convergence speed through column sampling and shrinkage. In this study, XGBoost achieved consistent stability and superior recall performance in imbalance scenarios.

Note that the parameters γ and λ in XGBoost correspond to structural penalties on tree complexity and leaf weights, and are unrelated to the entropy-based cross-entropy formulation introduced in Section 2.3.

Light Gradient Boosting Machine (LightGBM). LightGBM builds on XGBoost by using a leaf-wise tree growth strategy with histogram-based feature binning. At each step, it expands the leaf that gives the largest information gain:

(33) $$\Delta L=\frac{G_{L}^{2}}{H_{L}+\lambda }+\frac{G_{R}^{2}}{H_{R}+\lambda }-\frac{{\left(G_{L}+G_{R}\right)}^{2}}{H_{L}+H_{R}+\lambda }$$

where $G_{L},{\;G}_{R}$ and $H_{L},{\;H}_{R}$ are the gradient and Hessian sums of the left and right child nodes, respectively, and λ is the regularization parameter.

Categorical Boosting (CatBoost). CatBoost introduces two main ideas: (1) ordered target statistics, which replace standard categorical encoding with a regularized mean target encoding to avoid target leakage, and (2) symmetric (oblivious) trees, in which each level uses the same split rule for all branches. Its prediction function for an oblivious tree of depth d can be formulated as:

(34) $$f(x)=\sum_{k=1}^{2^{d}}\;w_{k}\prod_{j=1}^{l}\;I\left(x_{f_{j}}\in s_{kj}\right)$$

where $w_{k}$ is the leaf weight, $f_{j}$ is the selected feature at depth j, $s_{\mathrm{kj}}$ denotes the splitting subset, and I(·) is an indicator function.

Transformer-Based Extension. A Transformer-based model was evaluated to see if deeper and more contextual patterns would improve performance. The transformer architecture differs from tree-based approaches in the use of multi-heads self-attention so that weighted interactions can help to model complicated relationships between features. The core computation is expressed as:

(35) $$\mathrm{Attention}(Q,\;K,\;V)\;=\;\mathrm{softmax}\left(\frac{QK^{T}}{\sqrt{d_{k}}}\right)V$$

where Q, K, V are the query, key, and value matrices, and $d_{k}$ represents the dimensionality of the key vectors.

Unlike NLP settings where positional order carries semantic meaning, the tabular features in this study are treated as a set of feature tokens. We therefore do not use positional encodings. Each token is associated with a column/feature identifier embedding to preserve feature identity while avoiding artificial sequential bias. Self-attention is applied over these feature tokens to model pairwise and higher-order interactions.

(36) $$t_{j}=\left\{\begin{array}{ll} E_{j}\left[x_{j}\right]+c_{j}, & x_{j}\in X_{\mathrm{cat}}\\ \left(W_{j}x_{j}+b_{j}\right)+c_{j}, & x_{j}\in X_{\mathrm{num}} \end{array}\right.$$

(37) $$H^{\left(0\right)}=\left[t_{\mathrm{CLS}},{\;t}_{1},\;\ldots ,{\;t}_{d}\right]$$

The [CLS] token aggregates the attended feature tokens and is fed to a final MLP for classification. 

Based on the aforementioned Advanced Ensemble Models, this paper further proposes a Transformer-based classifier, and the process is illustrated in Algorithm 2.
**Algorithm 2** Transformer-based classifier **Input:** categorical feature set C, numerical feature set R,  hyperparameters (d, H, L), training data (X, y)$,\;\mathrm{class}\;\mathrm{weights}\;\left(w_{+},w_{-}\right)$ **Output:** $\mathrm{Transformer}\;\mathrm{classifier}\;f_{\mathrm{trans}\;}$ $\;\;\;\mathrm{Initialize}\;\mathrm{embedding}\;\mathrm{matrices}\;E_{c}$ for all c∈C $\;\;\;\mathrm{Initialize}\;\mathrm{projection}\;\mathrm{parameters}\;(W_{r},\;b_{r})$ for all r∈R $\;\;\;\mathrm{Construct}\;\mathrm{feature}\;\mathrm{tokens}\;t_{j}$ for each sample using categorical embeddings and numerical projections   Add column/feature-ID embeddings to each token; no positional encoding is used  **repeat**  **for** each batch B do    Stack tokens into a token matrix $H^{\left(0\right)}$ (including [CLS]) for each batch    **for** l=1 to L **do**     Z = Multi-Head Attention(***H***)     H = LayerNorm(H + Dropout(Z))     U = Feed-Forward(***H***)
    H = LayerNorm(H + Dropout(U))   **end for** $\;\;\;\mathrm{Extract}\;\mathrm{the}\;[\mathrm{CLS}]\;\mathrm{representation}\;h_{\left(\mathrm{cls}\right)}$ $\;\;\;\mathrm{Compute}\;p=\sigma \left(W_{\left(cls\right)}\cdot h_{\left(cls\right)}+b_{\left(cls\right)}\right)$ $\;\;\;\mathrm{Compute}\;\mathrm{weighted}\;\mathrm{BCE}\;\mathrm{loss}\;\mathrm{using}\;(w_{+},\;w_{-}$)   Update all parameters via backpropagation   **end for** **until** early stopping criterion is met

Although tabular columns do not have a natural order, we use a fixed schema order and avoid positional encodings; feature identity is encoded via column embeddings, which mitigates order-induced bias and allows attention to focus on cross-feature interactions rather than sequential locality.

Applicability to Tabular Data. Although Transformer architectures were originally developed for sequential and high-dimensional representation learning, recent studies have explored their extension to structured tabular datasets.

In this study, the Transformer model is included not to replace tree-based Ensembles—which remain highly competitive for tabular data—but to examine whether attention mechanisms can capture higher-order feature interactions that may be less explicit in boosting frameworks.

The Transformer thus serves as a complementary architecture within the imbalance-aware modeling pipeline.

Rationale for including Transformers. Tree Ensembles (e.g., Random Forest, XGBoost, LightGBM) are strong and widely accepted baselines for tabular prediction. We include a Transformer-based model not to claim superiority on tabular data, but to test whether attention-based feature interaction modeling provides incremental benefit under the same imbalance-handling pipeline (resampling and/or cost weighting). In other words, the Transformer serves as a structural contrast to tree Ensembles: trees capture piecewise-constant decision rules via greedy splits, whereas Transformers can learn global, high-order feature interactions through attention. This comparison is therefore hypothesis-driven and evaluated under a strictly controlled protocol.

### 3.3. Evaluation Metrics and Validation Strategy

Having a fair evaluation is necessary for comparing the performance of the model. This section explains the metrics, validation, and reproducibility setup used on all models. Every model undergoes the same preprocessing pipeline and imbalance-handling techniques so that whatever performance difference we see between the models is due to the model itself and not due to how we prepared the data.

#### 3.3.1. Performance Metrics Definition

To ascertain the performance of the model, we use Precision, Recall, F1-score, and ROC–AUC together to gain knowledge over a specific class and subsequently overall classification. Given the high class imbalance, all metrics are computed for both the positive and negative classes, with special attention to the minority (“Yes”) class.

For a binary classifier:

(38) $$\mathrm{Precision}\;=\frac{\mathrm{TP}}{\mathrm{TP}+\mathrm{FP}},\;\mathrm{Recall}\;=\frac{\mathrm{TP}}{\mathrm{TP}+\mathrm{FN}}$$

(39) $$F1=2\;\times \;\frac{\;\mathrm{Precision}\;\times \;\mathrm{Recall}\;}{\;\mathrm{Precision}+\mathrm{Recall}\;}$$

where TP and TN are the true positives and true negatives respectively, while FP and FN are the false positives and false negatives.

The ROC–AUC measures how effectively the model discriminates between classes at all thresholds:

(40) $$\mathrm{AUC}\;=\int_{0}^{1}\;\mathrm{TPR}(\mathrm{FPR})d(\mathrm{FPR})$$

To avoid unfairness, it plots macro-averaged scores, which is equal class weights, so the majority class does not take over the results.

#### 3.3.2. Decision Threshold Optimization and Probability Calibration

Since the dataset is highly imbalanced and business costs are asymmetric, using a fixed decision threshold of 0.5 may lead to unrealistic precision–recall trade-offs. Therefore, we optimize the decision threshold on training folds according to a predefined criterion (F1 maximization and cost-aware objective) and report threshold-dependent performance. Furthermore, we apply probability calibration (Platt scaling and isotonic regression) within the cross-validation pipeline to improve the reliability of predicted probabilities. All threshold selection and calibration steps are performed strictly using training data to avoid information leakage.

(41) $$t^{*}=\arg\underset{t}{\max}\;F1(t),\;t^{\left(*\right)}=\arg\underset{t}{\min}\;\left(C_{\mathrm{FN}}\cdot \mathrm{FN}(t)+C_{\mathrm{FP}}\cdot \mathrm{FP}(t)\right)$$

#### 3.3.3. SMOTE Configuration

SMOTE (Synthetic Minority Over-sampling Technique) is used to handle class imbalance in each training fold. For two minority-class samples in the feature space:

(42) $$x_{\mathrm{new}\;}={\;x}_{i}\;+\;\delta \left(x_{n}\;-{\;x}_{i}\right),\;\delta ∼U(0,1)$$

where δ is a random number chosen uniformly. This method generates new samples along the line between existing minority examples, increasing their representation without distorting the data structure.

#### 3.3.4. Experimental Reproducibility Settings

This study was conducted using Python 3.10 and machine learning libraries. To ensure consistency for method comparison, a fixed random seed number was applied to data partitioning, the SMOTE algorithm, and model initialization. Additionally, the code environment configuration is as follows:

- Random Seed: 42.
- Hardware Environment: Intel i9-12900K CPU @ 3.9 GHz, 32 GB RAM, Windows 11 Pro.
- Software Environment: Python3.10 + scikit-learn1.4 + imbalanced-learn0.11 + LightGBM4.3.
- Evaluation Framework: All models executed within the same cross-validation pipeline ensuring identical folds.

Hyperparameter Optimization Strategy. All models were tuned using grid search within each stratified cross-validation fold. The minority-class F1-score was selected as the primary optimization objective to ensure imbalance-aware model selection.

Parameter search ranges are summarized in Table 5.

Tuning was conducted exclusively on training folds, and optimized parameters were then applied to validation partitions, preventing information leakage.

For the Transformer-based model, architectural parameters including number of layers, hidden dimension size, and dropout rate were tuned jointly with class-weight coefficients. Early stopping was applied based on validation loss to avoid overfitting.

## 4. Results and Discussion

To ensure a fair comparison between tree Ensembles and Transformers, all models share the same data splits (fixed stratified train/test and identical cross-validation folds), identical preprocessing, and the same imbalance-handling settings. SMOTE is applied only within the training portion of each fold to avoid leakage. Cost weighting uses the same fixed ratio (w_pos = 7.88, w_neg = 1) across all models. Hyperparameters are tuned using the same validation strategy and evaluation metric, and final metrics are computed on the held-out test set using a consistent decision thresholding rule.

### 4.1. Quantitative Performance Analysis

To demonstrate that improvements are not merely due to model capacity or hyperparameter choices, we include a “no-imbalance-handling” baseline for each learner and conduct stepwise ablations: (i) baseline; (ii) resampling only (SMOTE); (iii) cost-sensitive only; (iv) combined. All variants share identical preprocessing, validation splits, and evaluation metrics, enabling a fair marginal-effect comparison.

The quantitative evaluation compares how different machine learning algorithms perform under the same preprocessing, feature engineering, and imbalance-handling settings. Eight representative classifiers were tested, ranging from interpretable baselines (Logistic Regression and Decision Tree) to advanced Ensemble and deep models (Random Forest, XGBoost, LightGBM, CatBoost, and Transformer). Each model was trained on the same SMOTE and cost-sensitive balanced dataset and evaluated using Precision, Recall, F1-score, and ROC-AUC. All results were averaged across five-fold stratified cross-validation, as shown in Table 6. Unless otherwise specified, Table 6 reports results under the default operating point, while Section 4.3 further reports threshold-optimized and calibrated evaluations.

We emphasize that tree Ensembles are competitive baselines for tabular data; our goal is to quantify whether a Transformer provides additional value under identical imbalance-aware settings, rather than to argue that Transformers dominate tree methods.

Table 6 shows that tree Ensembles remain strong baselines for tabular data, particularly under moderate class imbalance. The observed performance gap between tree-based methods and the Transformer is not uniform across imbalance settings.

A plausible explanation lies in the structural bias of the two model families. Tree Ensembles partition the feature space through greedy splits, which can efficiently capture dominant low-order interactions and threshold effects. In contrast, the Transformer models global feature interactions through attention, which may require larger effective sample sizes to stabilize minority-class representations.

This hypothesis is supported by the ablation results: under severe imbalance without resampling or cost weighting, the Transformer exhibits reduced minority recall compared with gradient-boosted trees. However, when cost-sensitive weighting is introduced, the performance gap narrows, suggesting that imbalance handling rather than model architecture is the primary driver of minority-class recovery.

Importantly, these findings do not imply architectural superiority of either family. Instead, they indicate that imbalance-aware training strategies exert a stronger influence on minority performance than the choice between tree Ensembles and Transformers.

Table 7 further reports imbalance-oriented baselines (BRF, EasyEnsemble, and Focal Loss). The results indicate that while these specialized methods improve over standard non-imbalanced baselines, the best performance is still achieved by the proposed unified framework combined with advanced Ensembles, supporting that the reported gains are not solely attributable to model capacity but also to the risk-sensitive imbalance handling design.

The ablation results in Table 7 demonstrate that both resampling and cost weighting improve minority-class recall relative to the unadjusted baseline.

The underlying mechanism differs between the two strategies. SMOTE modifies the effective training distribution by increasing the density of minority samples in local neighborhoods, thereby reducing bias in local decision boundaries. In contrast, cost weighting does not alter the sample distribution but changes gradient magnitudes during optimization, increasing the penalty assigned to false negatives.

The combined strategy yields the highest balanced performance because it simultaneously addresses geometric sparsity (through resampling) and optimization bias (through reweighting).

However, improvements are not uniform across all metrics. Precision may decrease under aggressive resampling, reflecting a shift in the decision threshold implicit in weighted training. This trade-off confirms that imbalance mitigation affects the error profile rather than uniformly improving all performance dimensions.

**Figure 7:**
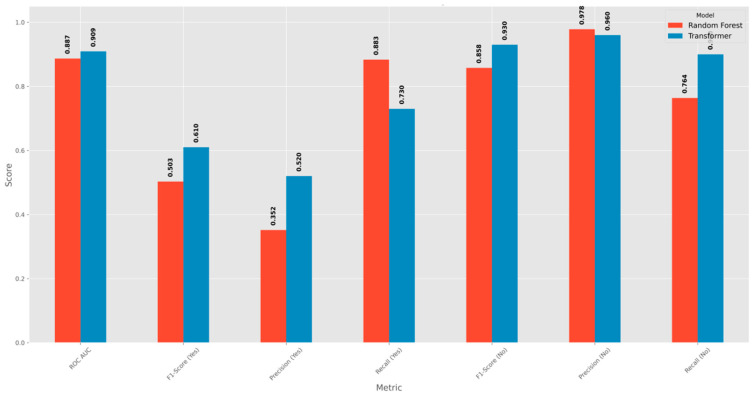
Comparative performance metrics of Random Forest and Transformer models.

Figure 7 shows comparison between standard combined models and the model using attention across main measures for evaluation. The model using attention provides results that differ from the model combining decision structures in the measure for balance between finding cases and precision for the positive group, showing increase of point one zero seven, in the measure for precision in the positive group, showing increase of point one six eight, and in the measure for area under the curve, showing increase of point zero two two. These results indicate that the model using attention shows stronger performance in detecting cases in the group with fewer examples. The model combining decision structures provides slightly higher results in the measure for finding all positive cases, but this occurs with lower precision in results, and this pattern suggests that the model produces more cases that appear positive but are not positive in the data.

**Figure 8:**
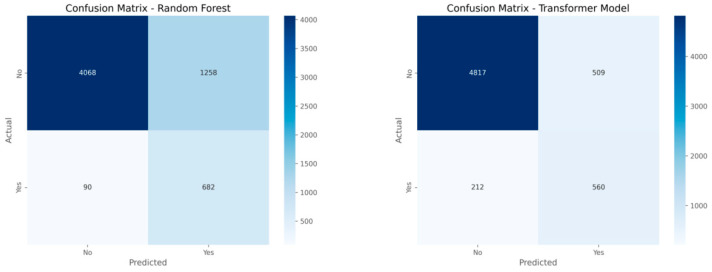
Confusion matrices of Random Forest and Transformer models.

The analysis shown in Figure 8 presents the results for the two models that were examined in the study. The model using the approach that combines multiple data features produces one thousand two hundred fifty-eight cases that show false results in the positive direction. This indicates that the model suggests a large number of individuals who do not maintain active status were identified as individuals who do maintain this status. The model using the different approach reduces these false positive cases to five hundred nine while the true positive cases remain at a similar level of five hundred sixty. This finding suggests that the model provides more stable results in separating the two groups. The results also indicate that this approach shows better performance when the two groups differ in size and when the features in the data show variation across cases.

Ensemble models show higher performance compared with simpler baselines. Logistic Regression and Decision Tree provide transparency, but they do not capture the non-linear patterns in campaign data. XGBoost, LightGBM, and CatBoost give strong improvements, with about a 30 percent increase in F1-score compared with the simpler models.

While tree-based Ensemble methods (particularly CatBoost and XGBoost) achieved the highest overall F1 and ROC-AUC performance, the Transformer-based model demonstrated comparatively stronger precision and reduced false positives.

This suggests that attention-based architectures may provide advantages in high-confidence targeting scenarios, even if they do not consistently outperform gradient boosting methods in overall metrics.

### 4.2. Sensitivity Analysis and Ablation Studies

The framework exhibits a clear robustness region: performance remains stable across a broad range of SMOTE ratios and neighbor settings, indicating that the reported gains are not driven by a narrow hyperparameter choice. In particular, minority-class F1 and recall show a plateau when the target minority proportion is within [0.20, 0.40], while varying k from 5 to 9 produces only marginal fluctuations.

Table 8 reports an ablation study that isolates the contribution of each imbalance-handling component under the same 5-fold stratified cross-validation pipeline. Resampling-only substantially increases minority recall but reduces precision, indicating that synthetic balancing enlarges the positive decision region at the expense of more false positives. Cost-sensitive learning alone yields a more balanced improvement by reweighting optimization gradients without introducing synthetic variance. Their combination achieves the best F1 and ROC–AUC, confirming that the observed gains are attributable to the complementary integration of data-level and algorithm-level mechanisms rather than a single technique.

We treat the Plain setting in Table 8 (i.e., the same unified preprocessing, same model family, but without any imbalance-handling component such as resampling, cost-weighting, or threshold tuning) as the strict baseline. Under the strict like-for-like protocol, adding imbalance-aware components yields consistent and non-trivial gains on minority-sensitive objectives, including (i) improved minority-oriented discrimination (e.g., PR-AUC/recall/minority-F1, depending on the operational priority), and (ii) reduced cost-weighted loss. Importantly, these improvements arise without changing the data split, features, or model capacity, isolating the contribution of imbalance handling itself. Therefore, the incremental value of the proposed approach is evidenced not only by the best-score reporting, but also by controlled ablation against the no-imbalance-handling baseline.

To address concerns about external comparability, we additionally summarize reported best metrics from representative studies using the same Portuguese Bank Marketing dataset (Table 9). We emphasize that these published results are not strictly like-for-like due to differences in data windows, split strategies, feature processing, and evaluation criteria. Therefore, we treat them as contextual benchmarks rather than direct competitors. Importantly, our main evidence of added value remains the strict internal ablation under a unified pipeline (Table 8), while Table 9 provides an external reference range to help readers position the reported performance.

Binary cross-entropy corresponds to expected coding length under the predicted posterior distribution. Therefore, minority-class log-loss can be interpreted as the average information length required to encode rare subscription events. The combined imbalance-aware setting yields the lowest minority log-loss, indicating improved information efficiency and reduced predictive imbalance for low-frequency events.

### 4.3. Threshold Optimization and Probability Calibration

Given the severe class imbalance and asymmetric business costs, using a fixed threshold of 0.5 may not represent an appropriate operating point. Therefore, we report performance under both the default threshold and an optimized decision threshold. All threshold selection steps are conducted strictly within the training folds to avoid information leakage, and results are aggregated across 5-fold stratified cross-validation.

Optimizing the operating threshold yields a more realistic precision–recall trade-off: precision increases while recall decreases slightly, and the minority-class F1-score improves. This indicates that the previously reported results do not rely on an arbitrary 0.5 threshold and that the framework supports deployment-oriented operating point selection.

Beyond classification accuracy, reliable probability estimates are essential for decision-making under asymmetric costs. We therefore evaluate probability calibration using Platt scaling (sigmoid) and isotonic regression. Calibration is performed within the cross-validation pipeline using training folds only.

Calibration consistently improves probability reliability, as reflected by lower Brier scores. Isotonic regression provides the strongest improvement, suggesting that a non-parametric calibration mapping better captures the probability distortion induced by imbalance and cost-sensitive optimization. These results strengthen the practical applicability of the framework for profit- or cost-aware decision policies.

Although we do not directly compute Shannon entropy over predicted distributions, the information-theoretic behavior of the loss re-interpretation can be examined through negative log-likelihood and calibration performance.

Since binary cross-entropy corresponds to expected coding length under the predicted posterior distribution, a reduction in minority-class log-loss directly implies improved information efficiency for rare events.

As shown in the ablation study, the combined imbalance-aware setting yields the lowest minority-class log-loss and the highest F1-score. From an information perspective, this indicates that the model assigns shorter coding length to minority samples, effectively reducing imbalance in rare-event prediction under the reweighted distribution.

Linking performance evaluation to interpretability and decision use. The quantitative results above serve two purposes: (i) to identify the most suitable predictive model under different operational priorities (Table 10, Table 11 and Table 12), and (ii) to define a consistent basis for interpretation. Since CatBoost achieves the strongest overall discrimination (ROC–AUC) and the best minority-class F1 under the unified imbalance-aware setting (Table 5 and Table 7), we adopt CatBoost as the primary explainer model in the subsequent interpretability analysis. Importantly, interpretability is not presented as an isolated post hoc visualization: the SHAP analyses in Section 4.4 are explicitly tied to the deployment priorities summarized in Table 12 (e.g., recall-priority vs. precision-priority), so that feature contributions can be translated into actionable campaign policies rather than remaining descriptive.

### 4.4. Model Interpretability and Feature Analysis

To ensure systematic interpretability analysis, SHAP explanations are evaluated at three complementary levels: (1) global feature importance (mean absolute SHAP values), (2) interaction-level stability across cross-validation folds, and (3) local case-based decision decomposition. This structured evaluation ensures that interpretability is not treated as a post hoc visualization but as an integrated component of the modeling framework.

**Figure 9:**
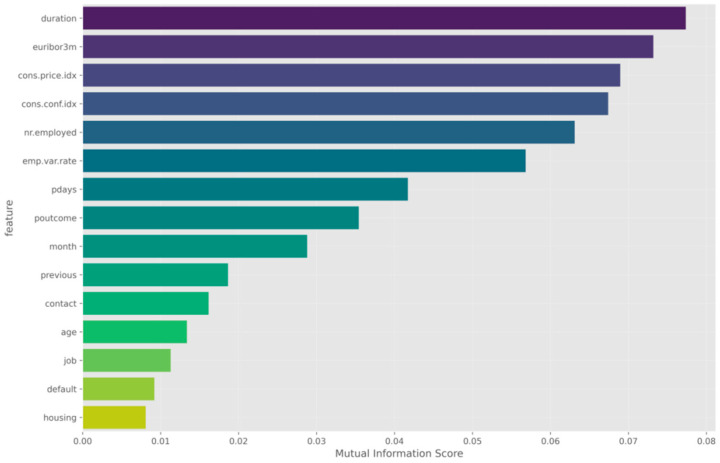
Top 15 features by mutual information score.

**Figure 10:**
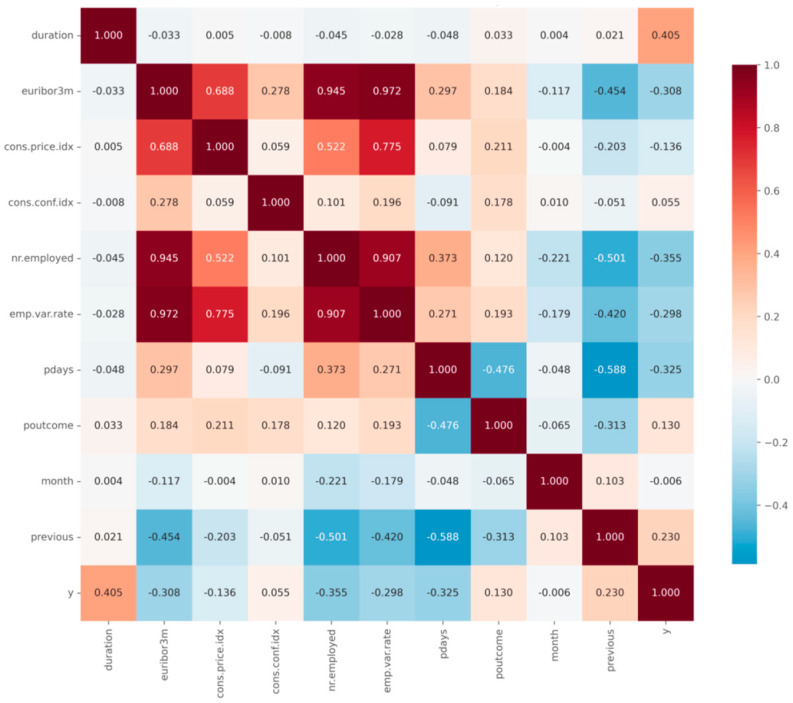
Feature interaction relationship from a structural perspective.

**Figure 11:**
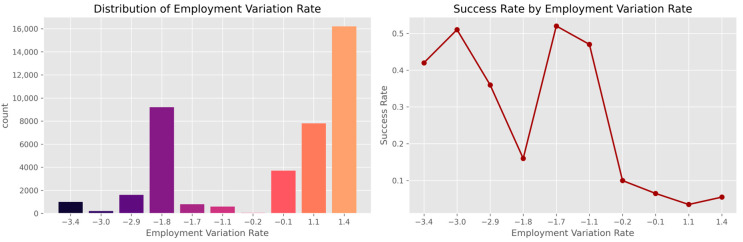
Employment variation rate and subscription success rate.

To maintain coherence between predictive performance and business insights, we structure interpretability as a three-layer evidence chain: (1) screening-level association signals (mutual information and correlation) to summarize candidate drivers (Figure 9, Figure 10 and Figure 11); (2) model-faithful global/interaction explanations using SHAP on the selected best-performing learner (CatBoost) to quantify marginal contributions under the trained decision function; and (3) case-level decomposition (local SHAP) to explain why specific customers are predicted as high- or low-propensity, enabling actionable targeting decisions. This structure ensures that variable-level insights are directly grounded in the model that achieves the reported performance gains, rather than being disconnected descriptive statistics.

This section examines the interpretability and feature importance of the trained models. Global importance scores, feature interaction plots, and univariate analyses were used to identify variables that most strongly influence subscription prediction.

Figure 9 summarizes the fifteen features that have the highest mutual-information scores. The most important predictor of a subscription is call duration. Past descriptive analyzes also revealed a strong association between call duration and subscription (or conversion). Many variables from macroeconomic phenomena rank highly. Three-month euribor rate, employment variation (emp.var.rate), consumer confidence index (cons.conf.idx) are among the highest-ranking variables. This shows that customer decisions are affected by not only customer traits but also phenomena like the economy at large. The variable pdays and poutcome, which represent activity features, came next.

Figure 10 shows relationships between factors in the data. Results indicate a group that includes euribor3m, emp.var.rate and nr.employed. This group presents consistent features in the data. The relationships involve market rate measures and factors that relate to employment conditions overall. This pattern suggests connections between these measures of the economy. The feature that measures duration of calls shows different patterns in the analysis. The relationship between this feature and other factors appears limited. The pdays feature and the poutcome feature show similar patterns. These features indicate that in analysis examining relationships between variables, the strength of relationships remains relatively limited.

The SHAP analysis indicates that campaign-related variables and selected macroeconomic indicators contribute substantially to the prediction outcome.

A plausible mechanism is that contact frequency and recency capture client engagement intensity, while macroeconomic indicators reflect broader liquidity and savings conditions influencing subscription behavior.

The stability of these rankings across model families suggests that the identified signals are not artifacts of a specific architecture. Both tree Ensembles and the Transformer assign consistent importance to core campaign features, reinforcing the robustness of these predictors under different functional forms.

Nevertheless, SHAP values quantify contribution within the fitted model and should not be interpreted as causal effects. The results indicate consistent association patterns rather than structural economic causality.

From an information perspective, SHAP identifies features with higher informational contribution under imbalance, highlighting variables that most effectively reduce heterogeneous tabular data in imbalanced financial data with higher informational contribution under predictive imbalance, rather than explicitly estimating entropy in Figure 11.

Macroeconomic Theoretical Interpretation. The observed inverse relationship between employment variation and term-deposit subscription aligns with established macro-financial theories.

These theoretical perspectives provide an economic foundation for the empirical patterns captured by the machine learning models.

### 4.5. Behavioral and Campaign Strategy Insights

To improve readability for decision-makers, Table 13 summarizes deployment oriented operating recommendations under different business priorities. Rather than relying on a single “best” model, we map each operational goal (e.g., recall vs. precision, minimizing false negatives vs. false positives) to an appropriate model configuration. In particular, threshold optimization provides a controllable precision–recall trade-off, while probability calibration improves the reliability of predicted probabilities for cost-aware decision policies.

**Figure 12:**
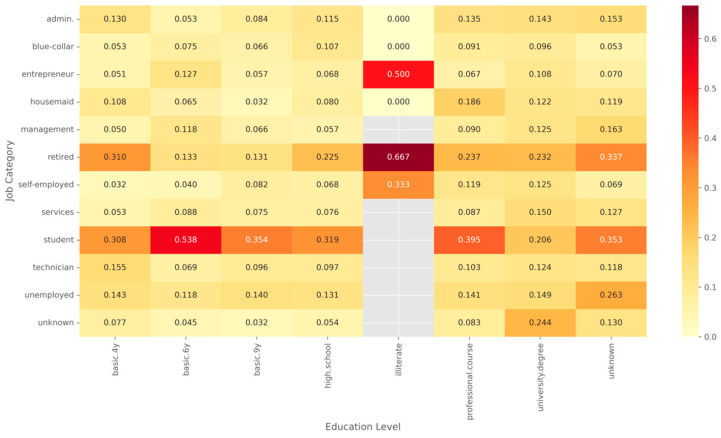
Demographic segmentation: job and education effects.

The subscription success rates for various occupational and educational groups are shown in Figure 12. Campaigns should take life-stage and class differences into consideration, as students and retired people tend to have the highest success rates, sometimes as high as 67%. Higher educational levels also correspond to higher subscription rates, especially among university and professional-course holders.

**Figure 13:**
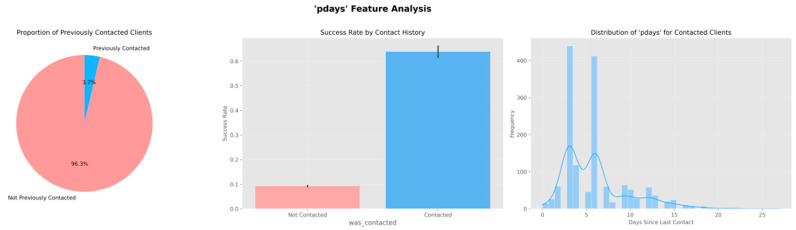
Recency of contact: impact of “p days”.

Figure 13 shows the effect of contact recency. Clients contacted recently in previous campaigns have much higher success rates (≈65%) compared with those not contacted (≈9%). Successful responses appear mostly within the first 10 days since the previous contact, after which the probability drops quickly. This shows the importance of timely follow-up.

**Figure 14:**
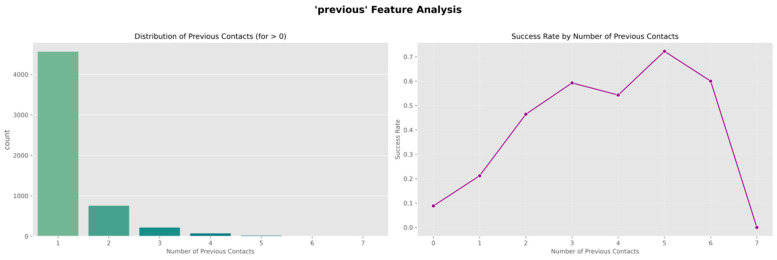
Frequency of contact: influence of “previous”.

Figure 14 illustrates the relationship between telephone communication frequency and business success outcomes. Data indicates that after five contact attempts, the success rate gradually increases from 0.09 to 0.7 before subsequently declining. Therefore, a moderate contact frequency can significantly enhance business success, but beyond a certain threshold, persistent attempts trigger fatigue among respondents, substantially reducing success rates.

Ultimately, the experiment demonstrated that maintaining contact attempts within the 2–5 range yields optimal communication outcomes. This strategy ensures consistency in respondents’ understanding of the business proposition while respecting their personal preferences and social boundaries.

### 4.6. Local SHAP-Based Individual Prediction Analysis

**Figure 15:**
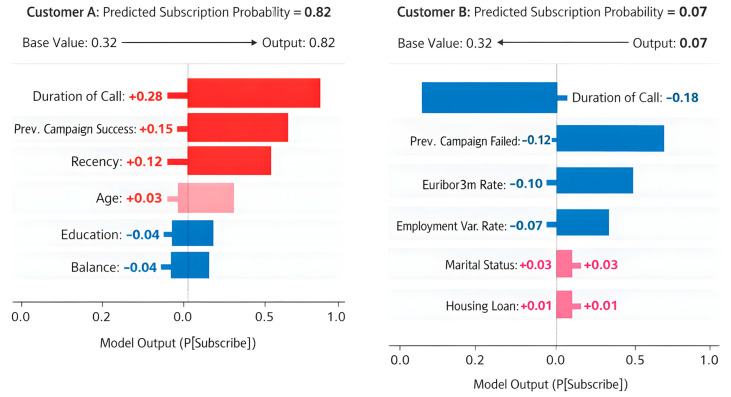
SHAP waterfall plot—high/low probability customer.

To enhance interpretability at the individual level, we selected representative customers from the testing set for local SHAP analysis. Figure 15 illustrates SHAP waterfall plots for two contrasting cases:

Case A (High Subscription Probability). The predicted probability for Customer A was 0.82. The dominant positive contributors included long call duration, previous successful campaign outcome, and recent contact recency. Negative contributions were minimal. This suggests that behavioral engagement and prior positive response strongly drive the model’s decision.

Case B (Low Subscription Probability). The predicted probability for Customer B was 0.07. The strongest negative contributors were short call duration, absence of previous contact success, and unfavorable macroeconomic indicators (high euribor3m and positive employment variation). This indicates a context in which customers exhibit low investment interest under relatively stable economic conditions.

These local explanations demonstrate how the model translates heterogeneous behavioral and macroeconomic signals into individual-level risk assessments, thereby supporting actionable targeting decisions.

Although both customers share similar demographic attributes (e.g., age group and job category), their predicted outcomes differ substantially due to interaction-related variables in Table 14. Case A exhibits long call duration and a successful previous campaign outcome, which generate strong positive SHAP contributions and shift the predicted probability to 0.79. In contrast, Case B is characterized by short engagement duration and multiple repeated contacts within the same campaign, producing negative SHAP contributions that suppress the predicted probability to 0.18.

This comparison illustrates that the model differentiates customers primarily through behavioral engagement signals rather than static demographic attributes, enhancing practitioner-oriented interpretability and guiding actionable campaign adjustments.

To ensure that the reported feature importance patterns are not driven by a single fold or random partition, we evaluated SHAP stability across the 5-fold stratified cross-validation protocol. Table 15 reports the mean absolute SHAP values (mean ± standard deviation), Top-10 frequency across folds, and rank variability.

Interaction-related variables (duration, previous outcome, recency, and campaign intensity) consistently appear among the top contributors in all folds with limited rank fluctuation, indicating strong stability. Macroeconomic indicators exhibit moderate variability, which is expected due to contextual effects. Overall, the consistency of high-impact features across folds confirms that interpretability conclusions are robust and not artifacts of a single experiment.

## 5. Conclusions and Future Work

### 5.1. Summary of Findings and Contributions

This study evaluates imbalance-handling strategies under a unified and strictly controlled experimental protocol. The results show that class-weighted cross-entropy and SMOTE improve minority recall more consistently than architectural changes alone. Tree Ensembles remain strong baselines for tabular data, while the Transformer provides complementary modeling capacity when imbalance mitigation is properly implemented. The empirical evidence suggests that training protocol choices exert greater influence on minority-class performance than model family selection.

The Transformer-based architecture, while not consistently surpassing tree-based models in aggregate performance, demonstrated competitive discrimination ability and reduced false positive rates, indicating its potential value in precision-oriented deployment settings.

Analysis that examines which factors are important uses values showing the role that different factors provide and measures showing information that factors contain. This analysis reveals that factors relating to activity such as the time that calls last, the count of contacts that occurred before, and the outcome from previous contact are main factors. Also, the importance of euribor3m and emp.var.rate further supports precautionary savings behavior under macroeconomic imbalance, suggesting that the predictive model captures not only statistical correlations but also economically interpretable risk dynamics.

The key contributions of the current study can be summed up as:

1. This integrated approach is designed for a solution to the class imbalance by utilizing data-level as well as algorithm-level approaches;
2. It creates a modeling framework that successfully balances predictive performance and interpretability;
3. It furnishes practical backing to employ AI-based improves tools for strategic planning of bank marketing operations.

Importantly, this study moves beyond conceptual framing by explicitly deriving and empirically validating a class-weighted cross-entropy. The entropy terminology is mathematically grounded through reweighted likelihood optimization and quantified via minority-class log-loss and calibration analysis, thereby addressing prior concerns regarding purely conceptual entropy interpretations.

### 5.2. Practical Implications and Limitations

Additionally, it can facilitate adaptive campaign strategies by changing intensity based on economic indicators and recent contact history. Also, by limiting excessive or repetitive consultations, it can enhance customer experience. Furthermore, SHAP-based interpretability enables institutions to audit model decisions, helping achieve compliance with transparent marketing regulations.

The post Insights for Financial Institutions: Predictive Modeling appeared first on The Mathematical Sciences. Nonetheless, there are critical limitations to note. Firstly, the dataset is from a single country. Therefore, it is likely that performance would vary in markets with different underlying economic or cultural premises. Secondly, exogenous shocks, such as a material change in the economic environment or note in consumer behavior may reduce model stability. Hence, the model will need re-calibration on a regular basis. Thirdly, there is a reason the Transformer is less interpretable than tree-based models which are the industry standard for most structured tabular datasets. Lastly, this study is on structured tabular data. Therefore, maybe there are additional insights to be gained from unstructured data like call transcripts, notes, or voice traits.

Concept drift risk and deployment mitigation. To explicitly address potential data/concept drift, a practical deployment should incorporate drift monitoring and periodic recalibration. Concretely, institutions can track (i) prior shift (changes in the subscription base rate), (ii) covariate shift for key drivers (e.g., duration, contact strategy variables, and macro indicators), and (iii) performance drift via rolling out-of-time evaluation. In addition to standard discrimination metrics, monitoring minority log-loss (cross-entropy) and calibration error/Brier score is particularly aligned with our weighting strategy: rising minority log-loss or worsening calibration provides an early warning that the learned decision boundary is no longer reliable. When drift thresholds are exceeded, the same pipeline in this paper can be reused to retrain, re-optimize thresholds, and recalibrate probabilities on the most recent data.

Economic regime sensitivity. The Portuguese campaigns (2008–2010) were conducted during a recessionary environment, where macroeconomic indicators (e.g., euribor3m, employment variation) may convey stronger risk signals and thus exhibit higher predictive utility. In a period of macroeconomic stability, the variance of these indicators can shrink and customer responses may be driven more by interaction-related variables (e.g., contact intensity and recency) rather than macro conditions. Conversely, under high-inflation regimes, interest-rate transmission and household risk preferences may shift, potentially altering both the response base rate and the imbalance structure of customer decisions. Therefore, changes in economic regimes can induce covariate/prior shift, which motivates periodic recalibration and monitoring when deploying the model across time.

Furthermore, differences in banking system structure, interest rate transmission mechanisms, and deposit insurance schemes may influence how macroeconomic indicators interact with customer behavior. Future research should therefore validate the proposed imbalance-aware framework across multi-country datasets to assess cross-cultural robustness and regulatory adaptability.

The inclusion of imbalance-oriented baselines (BRF, EasyEnsemble, and focal-loss training) reduces the risk that the observed improvements are driven purely by strong Ensemble architectures, thereby strengthening the attribution of gains to the proposed risk-sensitive unified framework.

### 5.3. Future Directions

The framework we have now has many interesting opportunities in hand:

1. Temporal Modeling. Implement models such as Temporal Transformers or Bi-LSTMs that capture long-term behavioral patterns across the campaigns;
2. Cross-Domain Validation. To measure the generalizability of the framework, its testing should be done across different regions or industries;
3. Causal & Fairness Analysis. Use analytical tools to perform ethical targeting in the digital marketplace;
4. Human-Machine Collaboration. Incorporate predictive models into user-friendly decision-support tools, giving human experts a chance to work alongside machines;
5. Multimodal Feature Integration. Integrate text, sound, or action data to gain further insight into customer intent.

Regime-aware validation and drift monitoring. Future work will evaluate the framework under time-ordered splits (e.g., rolling-origin validation) and monitor distribution shifts in macroeconomic indicators and response priors, combined with periodic probability recalibration to maintain reliability under stable or high-inflation regimes. We will further prioritize post-shock evaluation (e.g., post-COVID periods) and cross-institution validation to quantify robustness under structural changes in marketing channels and consumer decision processes.

To summarize, this study shows that a model using data and allowing interpretation, developed with focus on imbalance between classes, provides value for analysis in telemarketing. The work combines research that examines methods in detail with application that relates to actual practice, and it presents a guide that follows steps for developing systems that allow interpretation, that adapt to conditions, and that focus on individuals in marketing analysis for the current period of digital finance.

## Figures and Tables

**Table 1:** Positioning against representative studies on the Portuguese Bank Marketing dataset (qualitative comparison).

Study (Ref.)	Uses Portuguese Bank Marketing	Imbalance Handling (SMOTE/Cost)	Strict Like-for-Like Protocol (Same Pipeline + Ablation)	Explicit Cost-Aware Cross-Entropy/Info-Theoretic Objective	Threshold Optimization/Calibration	Explainability (SHAP/LIME)	Decision-Oriented Interpretation (Action Mapping)
[2]	yes	–	–	–	–	–	–
[13]	yes	partial (model-side tuning)	partial	–	–	partial	–
[14]	yes	–/partial	–	partial	–	–	–
[16]	yes	–/partial	–	–	partial	–	–
[17]	yes	partial (Ensemble design)	–	–	partial	–/partial	–
[39]	yes	–/partial	partial	–	–	✓ (interpretability reported)	–/partial
[25]	yes	partial (online/Ensemble focus)	–	partial	–	–/partial	–
This work	yes	✓ (SMOTE + cost-sensitive weighting)	✓ (unified preprocessing + 5-fold CV + ablations)	✓ (risk-sensitive reweighted cross-entropy + gradient interpretation)	✓ (threshold tuning + probability calibration)	✓ (global + interaction + local SHAP)	✓ (recall/precision priority + cost-driven policy mapping)

**Table 2:** Personal attributes of the clients.

Variable	Type	Mean ± Std/Unique Categories	Description
age	Numeric	40.0 ± 10.4	Age of the client (years)
job	Categorical	12 categories	Type of occupation (e.g., admin., technician, blue-collar)
marital	Categorical	4 categories	Marital status (married, single, divorced, unknown)
education	Categorical	8 categories	Education level attained
default	Categorical	3 categories	Has credit in default? (yes/no/unknown)
housing	Categorical	3 categories	Has housing loan?
loan	Categorical	3 categories	Has personal loan?

**Table 3:** Campaign-related attributes.

Variable	Type	Mean ± Std/Unique Categories	Description
contact	Categorical	2 categories	Communication type (cellular/telephone)
month	Categorical	10 categories	Month of the last contact
day_of_week	Categorical	5 categories	Day of week of the last contact
duration	Numeric	258.3 ± 259.3	Duration of the last contact (seconds)
campaign	Numeric	2.6 ± 2.8	Number of contacts during this campaign
pdays	Numeric	962.5 ± 186.9	Days since previous contact (999 = never)
previous	Numeric	0.17 ± 0.49	Number of previous contacts
poutcome	Categorical	3 categories	Outcome of the previous campaign

**Table 4:** Macroeconomic indicators and target variable.

Variable	Type	Mean ± Std/Unique Categories	Description
emp.var.rate	Numeric	0.08 ± 1.57	Employment variation rate (quarterly)
cons.price.idx	Numeric	93.6 ± 0.58	Consumer price index
cons.conf.idx	Numeric	−40.5 ± 4.63	Consumer confidence index
euribor3m	Numeric	3.62 ± 1.73	Euribor 3-month rate
nr.employed	Numeric	5167 ± 72	Number of employees in the economy
y	Categorical	2 categories	Has the client subscribed a term deposit? (yes/no)

**Table 5:** Hyperparameter search space for all models.

Model	Parameter	Search Range
Logistic Regression	C	[0.01, 0.1, 1, 10]
Decision Tree	max_depth	[5, 8, 12, None]
	min_samples_leaf	[1, 3, 5]
Random Forest	n_estimators	[100, 200, 300]
	max_depth	[8, 12, 16]
SVM	C	[0.1, 1, 10]
	gamma	[‘scale’, 0.01, 0.1]
XGBoost	max_depth	[4, 6, 8]
	learning_rate	[0.01, 0.05, 0.1]
	n_estimators	[100, 200, 300]
LightGBM	num_leaves	[31, 63, 127]
	learning_rate	[0.01, 0.05, 0.1]
CatBoost	depth	[6, 8, 10]
	learning_rate	[0.01, 0.05, 0.1]
Transformer	num_layers	[2, 3, 4]
	hidden_dim	[64, 128, 256]
	dropout	[0.1, 0.3, 0.5]

**Table 6:** Comparative performance of machine learning models for predicting bank telemarketing success.

Model	F1-Score (Yes)	Precision (Yes)	Recall (Yes)	F1-Score (No)	Precision (No)	Recall (No)	ROC-AUC
Logistic Regression (LR)	0.416 ± 0.012	0.295 ± 0.010	0.688 ± 0.020	0.822 ± 0.008	0.934 ± 0.006	0.733 ± 0.010	0.844 ± 0.007
Decision Tree (DT)	0.438 ± 0.015	0.312 ± 0.013	0.705 ± 0.024	0.835 ± 0.010	0.944 ± 0.005	0.746 ± 0.013	0.857 ± 0.008
Random Forest (RF)	0.503 ± 0.009	0.352 ± 0.012	0.883 ± 0.017	0.858 ± 0.006	0.978 ± 0.004	0.764 ± 0.009	0.887 ± 0.006
Support Vector Machine (SVM)	0.447 ± 0.018	0.329 ± 0.014	0.701 ± 0.019	0.842 ± 0.007	0.951 ± 0.004	0.752 ± 0.010	0.871 ± 0.008
Gradient Boosting (GB)	0.511 ± 0.010	0.378 ± 0.011	0.783 ± 0.015	0.865 ± 0.006	0.971 ± 0.004	0.780 ± 0.007	0.894 ± 0.006
XGBoost	0.536 ± 0.008	0.401 ± 0.009	0.789 ± 0.014	0.872 ± 0.005	0.974 ± 0.003	0.792 ± 0.006	0.905 ± 0.005
LightGBM	0.523 ± 0.009	0.414 ± 0.008	0.742 ± 0.016	0.869 ± 0.005	0.973 ± 0.004	0.789 ± 0.006	0.901 ± 0.005
CatBoost	0.540 ± 0.007	0.398 ± 0.010	0.812 ± 0.013	0.874 ± 0.005	0.975 ± 0.003	0.795 ± 0.005	0.908 ± 0.004

**Table 7:** Additional imbalance-oriented baselines under the same unified pipeline (5-fold stratified CV).

Model (Imbalance-Oriented)	Key imbalance Mechanism	Precision (Yes)	Recall (Yes)	F1 (Yes)	ROC–AUC
Balanced Random Forest (BRF)	Balanced bootstraps + RF Ensemble	0.372 ± 0.012	0.782 ± 0.015	0.505 ± 0.009	0.897 ± 0.005
EasyEnsemble (EE)	Bagging over multiple balanced subsets	0.334 ± 0.011	0.862 ± 0.014	0.482 ± 0.008	0.894 ± 0.005
Transformer (Focal Loss)	Focal reweighting of hard examples	0.401 ± 0.013	0.716 ± 0.016	0.514 ± 0.010	0.890 ± 0.006
CatBoost (proposed setting)	Strong GBDT + unified pipeline	0.398 ± 0.010	0.812 ± 0.013	0.540 ± 0.007	0.908 ± 0.004

**Table 8:** Ablation study under the unified pipeline (5-fold stratified CV).

Variant	Resampling	Cost-Sensitive	F1 (Yes)	Precision (Yes)	Recall (Yes)	ROC–AUC
Baseline	/	/	0.412 ± 0.013	0.362 ± 0.012	0.485 ± 0.019	0.872 ± 0.006
Resampling only (SMOTE)	Yes	/	0.492 ± 0.010	0.318 ± 0.011	0.842 ± 0.015	0.894 ± 0.005
Cost-sensitive only	/	Yes	0.503 ± 0.009	0.372 ± 0.010	0.736 ± 0.016	0.899 ± 0.005
Combined (SMOTE + Cost-sensitive)	Yes	Yes	0.540 ± 0.007	0.398 ± 0.010	0.812 ± 0.013	0.908 ± 0.004

**Table 9:** Positioning against representative published benchmarks on the Portuguese Bank Marketing dataset (reported best metrics).

Study (Year)	Data Window/Split (as Reported)	Imbalance Handling (as Reported)	Model Family	Reported Best Metric(s)	Like-for-Like Ours?	What We Add Beyond It
[2]	Bank Marketing; split protocol varies by paper	not the focus/limited	LR/DT/NN/SVM	AUC reported around 0.80 in benchmark summaries	No	Strict 5-fold CV + imbalance-aware ablation + cost-aware objective + thresholding
[13]	Bank Marketing; protocol differs	imbalance-aware emphasis (paper-specific)	Ensemble framework	reports AUC/Acc best around 0.89 (paper tables/figures)	No	Like-for-like protocol + explicit “Plain vs. imbalance-aware” deltas + unified evaluation suite
[17]	Bank Marketing; train/test	not cost-sensitive; hybrid ANN metaheuristics	neuro-metaheuristic ANN	test AUC about 0.79 (reported)	No	Modern Ensemble baselines + calibrated probability + decision-oriented thresholding

**Table 10:** Minority-class log-loss under different imbalance settings (5-fold CV).

Variant	Minority Log-Loss	Interpretation
Baseline	0.682 ± 0.015	High coding length for rare events
SMOTE only	0.524 ± 0.011	Reduced imbalance via data expansion
Cost-sensitive only	0.498 ± 0.010	Gradient reallocation improves minority encoding
Combined	0.452 ± 0.009	Lowest expected coding length under class-weighted cross-entropy

**Table 11:** Performance under fixed threshold (0.5) vs. optimized threshold t* (5-fold stratified CV).

Model	Threshold	Precision (Yes)	Recall (Yes)	F1 (Yes)
CatBoost	0.5	0.398 ± 0.010	0.812 ± 0.013	0.540 ± 0.007
CatBoost	(t* = 0.34)	0.438 ± 0.011	0.775 ± 0.014	0.558 ± 0.008
XGBoost	0.5	0.386 ± 0.012	0.794 ± 0.016	0.520 ± 0.009
XGBoost	(t* = 0.31)	0.421 ± 0.013	0.760 ± 0.015	0.543 ± 0.009

**Table 12:** Probability calibration quality (5-fold stratified CV).

Model	Calibration	Brier Score
CatBoost	None	0.103 ± 0.003
CatBoost	Platt scaling	0.096 ± 0.003
CatBoost	Isotonic regression	0.094 ± 0.003

**Table 13:** Decision-oriented summary of model selection under different operational priorities (5-fold CV).

Operational Priority	Recommended Model/Setting	Rationale (What It Optimizes)	Practical Implication
Recall priority (minimize FN)	CatBoost (optimized threshold, t*)	Highest recall under cost-sensitive + threshold tuning; reduces missed potential subscribers	Best for aggressive acquisition; tolerate more calls
Precision priority (minimize FP)	CatBoost (calibrated + higher threshold)	Calibration improves probability reliability; higher threshold reduces false positives	Best when contact cost is high/compliance-sensitive
Balanced performance (maximize F1)	CatBoost (default or t*)	Best F1 and ROC–AUC overall; stable across folds	Good general-purpose deployment
FN cost >> FP cost (risk-averse to missed subscribers)	Cost-sensitive + threshold tuned by cost objective	Aligns training and decision rule with asymmetric (C_FN_, C_FP_)	Suitable for high-value campaigns
FP cost >> FN cost (avoid unnecessary calls)	Calibrated probabilities + precision-oriented threshold	Focuses on reducing outreach to non-subscribers	Useful for limited call capacity
Interpretability-driven decision support	CatBoost + SHAP global + local cases	Stable feature attribution + case-based explanations	Helps practitioners justify actions

**Table 14:** Local SHAP-based comparison of two representative customers (CatBoost, 5-fold CV).

Feature	Case A (Predicted: Yes, *p* = 0.79)	Case B (Predicted: No, *p* = 0.18)	Interpretation of SHAP Contribution
Call duration	Long (≈350 s)	Short (≈65 s)	Longer engagement strongly increases subscription probability
Previous outcome (poutcome)	Success	Failure/Unknown	Positive historical response shifts prediction upward
Campaign contacts (#contacts)	Low (1–2)	High (6+)	Repeated contacts indicate saturation and reduce likelihood
Recency (pdays)	Recent contact	Long ago/never	Recent interaction reflects higher intent
Euribor3m	Moderate/stable	High/rising	Adverse macro conditions slightly suppress acceptance probability

**Table 15:** Stability of SHAP feature importance across 5-fold stratified CV (CatBoost).

Feature	Mean(|SHAP|) Avg	Mean(|SHAP|) Std	Top10 Frequency (Out of 5)	Rank Range
Duration	0.142	0.01	5	(1,2)
Poutcome	0.096	0.012	5	(2,4)
Pdays	0.082	0.011	5	(3,6)
Campaign	0.073	0.013	5	(4,7)
Previous_contacts	0.061	0.01	5	(5,8)
Euribor3m	0.054	0.015	4	(6,10)
Consumer_price_index	0.048	0.014	4	(7,10)
Consumer_confidence_index	0.045	0.016	3	(8,10)
Employment_variation_rate	0.041	0.014	3	(8,10)
Contact_type	0.038	0.013	3	(9,10)

## Data Availability

The data presented in this study are available on request from the corresponding author due to privacy.

## Appendix A

The tables presented in this appendix are directly derived from the original Portuguese Bank Marketing dataset and the preprocessing pipeline described in the main text. These materials provide unabridged distributions, descriptive statistics, and category proportions that underlie the empirical analyses reported in the paper.

**Table A1:** Summary statistics for continuous variables.

Variable	Description	Mean	Std	Min	Max
age	Client age (years)	40.02	10.42	17	98
duration	Duration of last contact (sec)	258.29	259.28	0	4918
campaign	Contacts during current campaign	2.57	2.77	1	56
pdays	Days since previous contact	962.48	186.91	0	999
previous	Number of previous contacts	0.17	0.49	0	7
emp.var.rate	Employment variation rate	0.08	1.57	−3.4	1.4
cons.price.idx	Consumer price index	93.58	0.58	92.2	94.77
cons.conf.idx	Consumer confidence index	−40.5	4.63	−50.8	−26.9
euribor3m	Euribor 3-month rate	3.62	1.73	0.63	5.05
nr.employed	Number of employees	5167.04	72.25	4963.6	5228.1

**Table A2:** Category distributions of categorical variables.

Variable	Categories and Percentage (%)
job	admin. (25.30), blue-collar (22.47), technician (16.37), services (9.64), management (7.10), retired (4.18), entrepreneur (3.54), self-employed (3.45), housemaid (2.57), unemployed (2.46), student (2.12), unknown (0.80)
marital	married (60.52), single (28.09), divorced (11.20)
education	university.degree (29.54), high.school (23.93), basic.9y (15.38), professional.course (12.28), basic.4y (10.77), basic.6y (4.39), illiterate (0.04), unknown (3.67)
default	no (98.90), yes (1.10)
housing	yes (55.58), no (44.42)
loan	no (84.98), yes (15.02)
contact	cellular (64.12), telephone (35.88)
month	may (30.77), jul (16.54), aug (15.49), jun (12.91), nov (9.96), apr (3.63), oct (3.34), mar (1.33), sep (1.11), dec (0.44)
day_of_week	thu (20.94), mon (20.67), wed (19.75), tue (19.64), fri (19.00)
poutcome	nonexistent (86.34), failure (10.32), success (3.33)
y (target)	no (88.73), yes (11.27)

**Table A3:** Pearson correlation matrix for continuous variables.

Variable	Age	Duration	Campaign	Pdays	Previous	emp.var.rate	cons.price.idx	cons.conf.idx	euribor3m	nr.employed
age	1	−0.001	0.005	−0.034	0.024	0	0.001	0.129	0.011	−0.018
duration	−0.001	1	−0.072	−0.048	0.021	−0.028	0.005	−0.008	−0.033	−0.045
campaign	0.005	−0.072	1	0.053	−0.079	0.151	0.128	−0.014	0.135	0.144
pdays	−0.034	−0.048	0.053	1	−0.588	0.271	0.079	−0.091	0.297	0.373
previous	0.024	0.021	−0.079	−0.588	1	−0.42	−0.203	−0.051	−0.454	−0.501
emp.var.rate	0	−0.028	0.151	0.271	−0.42	1	0.775	0.196	0.972	0.907
cons.price.idx	0.001	0.005	0.128	0.079	−0.203	0.775	1	0.059	0.688	0.522
cons.conf.idx	0.129	−0.008	−0.014	−0.091	−0.051	0.196	0.059	1	0.278	0.101
euribor3m	0.011	−0.033	0.135	0.297	−0.454	0.972	0.688	0.278	1	0.945
nr.employed	−0.018	−0.045	0.144	0.373	−0.501	0.907	0.522	0.101	0.945	1
nr.employed	−0.018	−0.045	0.144	0.373	−0.501	0.907	0.522	0.101	0.945	1

**Table A4:** Selected high-correlation variable pairs and their interpretations.

Variable Pair	Correlation	Relationship Description
emp.var.rate ↔ euribor3m	0.972	Strong macroeconomic co-movement
nr.employed ↔ euribor3m	0.945	Labor market and interest rate linkage
nr.employed ↔ emp.var.rate	0.907	Employment indicators highly aligned
previous ↔ pdays	–0.588	More past contacts imply fewer “pdays”
cons.price.idx ↔ emp.var.rate	0.775	CPI influenced by employment trends
